# Periodontal Disease and Edentulism Trends in Older Women of China and Association of Southeast Asian Nations, 1990 to 2033

**DOI:** 10.1016/j.identj.2026.109780

**Published:** 2026-07-24

**Authors:** Yilin Zhang, Xiaowei Dai, Yuan Tang, Jing Li, Fei He, Lei Zhao, Xin Xu

**Affiliations:** a School of Stomatology, Shandong Second Medical University, Weifang, Shandong, PR China; b Affiliated Hospital of Shandong Second Medical University, Weifang, Shandong, PR China; c School of Public Health, Shandong Second Medical University, Weifang, Shandong, PR China; d Hospital of Stomatology Xi’an Jiaotong University, Xian, Shanxi, PR China; e Rizhao Lanshan District People’s Hospital, Rizhao, Shandong, PR China

**Keywords:** Disease burden, Health policy, Geriatric dentistry, Disability‑adjusted life years, ASEAN, China

## Abstract

**Aims:**

To assess trends, co-occurrence patterns, and future projections of periodontal disease and edentulism burden among women aged ≥60 years in China and Association of Southeast Asian Nations (ASEAN) countries from 1990 to 2033.

**Methods:**

Using Global Burden of Disease 2023 data, we analysed age-standardized disability-adjusted life years (DALYs) for both conditions in China and 10 ASEAN countries. Quartile-based co-occurrence classification, frontier analysis against the Sociodemographic Index, and ARIMA models (2024-2033) were applied.

**Results:**

In 2023, periodontal disease DALY rates were highest in Thailand (183.93 per 100,000) and Indonesia (183.77); edentulism rates peaked in the Philippines (948.96) and Malaysia (811.45). From 1990 to 2023, edentulism declined across most countries (ASEAN aggregate: 709.39-641.96), while periodontal disease increased in Indonesia and Thailand. Frontier analysis showed edentulism burden plateaus at higher Sociodemographic Index levels. ARIMA projections indicate continued edentulism decline through 2033 (ASEAN: 636-586) but rising periodontal disease burden in China and Indonesia.

**Conclusion:**

The burden is shifting from tooth loss to chronic periodontal inflammation among aging women in China and ASEAN. Country-specific strategies prioritizing periodontal screening and integrated oral primary care are needed.

**Clinical relevance:**

These findings inform targeted oral health policies for older women, emphasizing the need to shift from tooth preservation alone to active periodontal management. Specifically, countries with rising periodontal burden should prioritize screening and non‑surgical therapy, while those with persistent high edentulism need expanded restorative and preventive coverage—particularly in rapidly developing regions where tooth retention is increasing but maintenance lags behind.

## Introduction

Oral disorders affect over 3.5 billion people worldwide, with periodontal disease and edentulism forming a causal continuum that often ends in tooth loss and seriously impacts systemic health and quality of life.^1^ From 1990 to 2021, the incidence of periodontal disease rose by 76.32% and edentulism by 93.56%, driven by population aging and disparities in oral healthcare access.^2^ Sex and age specific disparities are pronounced. Although males have higher overall rates, females have higher DALYs. Among women aged 60 years and older, periodontal disease burden has risen steadily, with a cumulative increase of 1.14% from 1990 to 2021.^3^ This trend reflects hormonal fluctuations, menopause related changes in oral immunity and bone metabolism, gendered healthcare seeking behaviour, and cumulative risk exposures.^4^ Older women face intersecting vulnerabilities including lifelong disease progression, menopausal changes, greater longevity, and often limited economic resources, making them uniquely susceptible. However, targeted research on this population remains limited.^5^

The 2023 GBD study provides the latest and most robust estimates, incorporating updated data and post pandemic demographic adjustments.^6^ DALYs are especially valuable for assessing nonfatal conditions like periodontal disease and edentulism, as they measure overall health loss from disability.^7^ A comparative analysis of China and ASEAN is strategically relevant. China accounts for 18.93% of global oral disease DALYs, the highest burden worldwide, with older women aged 60+ identified as a priority population. In ASEAN, which includes 10 nations at different development stages, prevalent cases rose over 50% and incident cases over 30% from 1990 to 2021, driven by population growth and aging. Periodontal disease is the second leading cause of oral disease burden in ASEAN, after untreated caries.^8^ Despite geographic proximity and some demographic similarities, China and ASEAN differ markedly in healthcare organization, oral health policies, and economic development. How these factors shape the oral disease burden in older women remains poorly understood.

Therefore, this study aimed to comprehensively compare the oral health burden from periodontal disease and edentulism among women aged ≥60 years in China and ASEAN countries from 1990 to 2023, using the latest GBD 2023 data with DALYs as the primary outcome. We applied frontier analysis to assess the minimum achievable burden across countries by socio demographic development level, and ARIMA models to project disease burden trends through 2033. By analysing temporal trends, burden distribution, and frontier efficiency, we sought to provide evidence for targeted oral health policies, resource allocation, and prevention strategies for this growing and vulnerable population in the China ASEAN region. The findings are expected to advance the global oral health agenda, especially amid population aging and the urgent need to reduce oral health inequities among older women in rapidly developing regions.

## Methods

### Data sources and study design

This study is a large-scale descriptive epidemiological analysis based on the GBD 2023 dataset, constituting a specific subanalysis focused on periodontal disease and edentulism among women aged 60 years and older in China and ASEAN countries. Data were obtained from the GBD 2023 repository via the Global Health Data Exchange (GHDx; http://ghdx.healthdata.org), which provides comprehensive estimates of incidence, prevalence, DALYs, and their temporal trends for 375 diseases and injuries globally.^6^ Inclusion criteria for countries or entities: All 10 ASEAN member states (Brunei, Cambodia, Indonesia, Lao PDR, Malaysia, Myanmar, Philippines, Singapore, Thailand, and Vietnam) and China were included. Inclusion criteria for years: The full time range from 1990 to 2023 was included. Inclusion criteria for outcomes: The age‑standardized DALY rate (ASDR) was included as the primary burden metric. The ‘ASEAN average’ reported throughout this manuscript refers to the population‑weighted average of the 10 ASEAN member states. All averages were calculated using population weights based on GBD 2023 population estimates for each year.^9^

In the GBD 2023 framework, ‘periodontal disease’ refers specifically to severe periodontitis, with definitions corresponding to the International Classification of Diseases, Ninth Revision (ICD‑9) codes 523.0 to 523.9, and Tenth Revision (ICD‑10) codes K05.0 to K05.6, while edentulism was defined using ICD‑10 code K08.2.^7^ This study focused exclusively on the DALYs as a burden metric. DALYs quantify overall health loss by summing the years of life lost due to premature mortality and years lived with disability, providing a comprehensive measure of the gap between current health status and an ideal health standard.

### Statistical analysis

#### Definition and regional division of co-occurrence characteristics of periodontal disease and edentulism based on DALY quartiles

To explore the co-occurrence characteristics of periodontal disease and edentulism as well as the differences in their spatial distribution across China and ASEAN countries, we categorized the ASDR rates of both conditions into four classification levels based on quartiles: low (less than 25th percentile), lower middle (25th to 50th percentile), upper middle (50th to 75th percentile), and high (greater than 75th percentile).^10^ If the DALY rate levels of both conditions in a country or territory were identical, that country or territory was classified as a consistent unit and all such units together formed a consistent area. If the periodontal disease DALY rate level was higher than that of edentulism in a country or territory, it was designated as a periodontal disease-dominant unit, and all such units formed the periodontal disease-dominant area. Similarly, if the edentulism DALY rate level exceeded that of periodontal disease, the country or territory was designated as an edentulism dominant unit, and all such units formed the edentulism dominant area. Consequently, the study area was divided into three regions, each representing a distinct pattern of co-occurrence of periodontal disease and edentulism.^11^

#### Frontier analysis

Frontier analysis is a robust quantitative method for exploring the relationship between disease burden and the Sociodemographic Index (SDI) and identifying opportunities for health improvement.^12^ This study applies a frontier model based on the ASDR to examine how periodontal disease and edentulism among women aged 60 years and older in China and ASEAN countries are influenced by the SDI. Unlike conventional models, frontier analysis captures the nonlinear relationship between the SDI and the burden of these oral conditions. It identifies key factors, such as income levels, healthcare access, and education, which significantly contribute to periodontal disease and edentulism, offering deeper insights into this complex relationship. This approach integrates locally weighted and polynomial regressions to construct smoothed frontiers, thereby evaluating each country’s potential for improvement by measuring the gap between its ASDR and the frontier for each year from 1990 to 2023.^13^

#### ARIMA model

For ARIMA projections, we included five populations: the ASEAN aggregate, China, Indonesia, Thailand, and Malaysia. Selection criteria were large population size to reduce prediction error, stable low volatility time series data for accurate model fitting, and regional representativeness for policy relevance. Smaller ASEAN members were excluded due to high data volatility, which would make forecasts clinically and epidemiologically meaningless.^14^ We followed a stepwise procedure: checked the ASDR time series for stationarity, applied differencing (d) as needed, examined ACF and PACF plots to identify autoregressive (p) and moving average (q) orders, and fitted candidate models (**p=0-5, d=0-2, q=0-5) using maximum likelihood estimation. The final model was selected by minimizing AIC and BIC. All analyses used R 4.3.2 with the forecast package, where auto.arima() selected the optimal model based on AICc. Model fit was evaluated using MAPE and RMSE on the training set.^15^ Of note, ARIMA models are purely statistical extrapolations based on historical patterns (1990-2023). These projections should be interpreted as trend based illustrations of what would happen if past patterns continued, not as deterministic predictions.

## Results

### Age‑standardized DALY rates of periodontal disease and edentulism in China and ASEAN countries, 2023

**Fig. 1:**
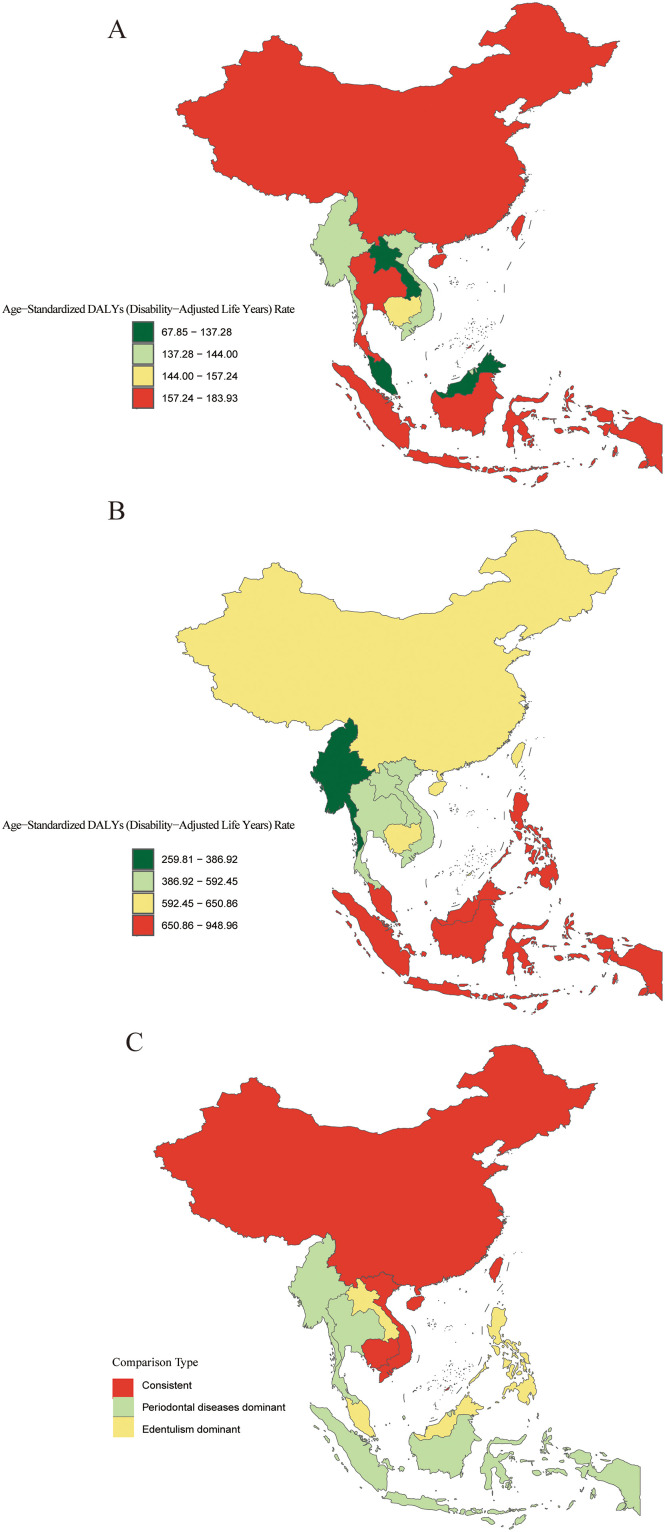
Age-standardized disability-adjusted life years (DALYs) rates of periodontal disease (A) and edentulism (B), and comorbidity (C) in China and ASEAN in 2023.

In 2023, age‑standardized DALY rates for periodontal disease in women ≥60 years (Figure 1A, Supplementary Table 1) were highest in Thailand (183.93; 95% UI: 69.23-355.41) and Indonesia (183.77; 69.74-376.78), followed by China (164.78; 61.39-323.63). Moderate rates (130-160) were seen in Malaysia (135.03; 47.05-257.03), Cambodia (149.71; 55.59-324.87), Vietnam (139.54; 47.85-276.43), Singapore (145.35; 50.71-280.84), Myanmar (144.00; 50.33-285.80), and Brunei (142.45; 48.62-286.23). Low rates (<100) were in Lao PDR (98.59; 35.61-223.93) and the Philippines (67.85; 23.44-132.05).

For edentulism (Figure 1B, Supplementary Table 2), rates were highest in the Philippines (948.96; 612.03-1289.16) and Malaysia (811.45; 518.46-1166.59), followed by Indonesia (660.54; 426.56-895.01), Cambodia (641.18; 417.13-915.79), China (619.35; 419.09-864.02), Thailand (592.45; 374.64-857.74), Vietnam (545.32; 344.82-775.48), Lao PDR (391.55; 242.62-566.75), Myanmar (382.28; 249.04-557.36), Singapore (318.65; 207.25-457.77), and Brunei (259.81; 168.52-384.42).

Based on quartile classification (Figure 1C, Supplementary Table 3), three co‑occurrence patterns emerged: periodontal disease‑dominant (Indonesia, Singapore, Myanmar, Brunei, Thailand), edentulism‑dominant (Malaysia, Lao PDR, Philippines), and consistent areas (Cambodia, Vietnam, China).

### Temporal trends in age‑standardized DALY rates of periodontal disease and edentulism, 1990 to 2023

**Fig. 2:**
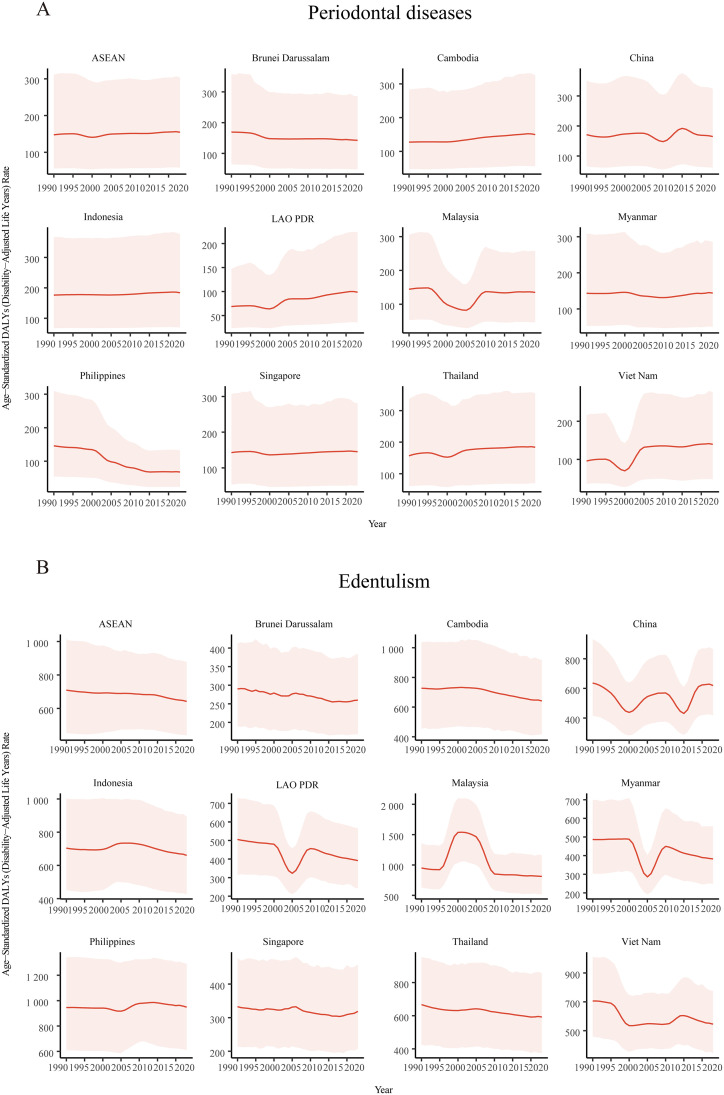
Trends in age-standardized disability-adjusted life years (DALYs) rates of periodontal disease (A) and edentulism (B) in China and ASEAN countries from 1990 to 2023.

From 1990 to 2023, the ASEAN age‑standardized DALY rate for periodontal disease in women ≥60 years increased from 147.65 to a peak of approximately 151 in 2007, fluctuated between 150 and 156, then declined to 154.60 in 2023. In China, the rate decreased from 170.99 to 164.78. In 2023, the highest rates were in Indonesia (183.77) and Thailand (183.93); lower rates in the Philippines (67.85), Malaysia (135.03), and Singapore (145.35). Myanmar, Vietnam, and Cambodia ranged from 139 to 152, near the regional average. Lao PDR rose from approximately 69 in the early 1990s to 98.59 in 2023, still below average (Figure 2A, Supplementary Table 4).

For edentulism, the ASEAN regional rate fluctuated between 640 and 710 from 1990 to 2023, declining from 709.39 to 641.96. China declined from 636.17 to 619.35. The Philippines and Malaysia had the highest burdens, with rates remaining above 940 and between 800 and 950, respectively. Moderate rates (400-750) were seen in Cambodia, Indonesia, Thailand, Vietnam, Lao PDR, and Myanmar; low rates (250-330) in Singapore and Brunei. Most countries showed decreasing trends (China, Thailand, Vietnam, Lao PDR, Myanmar, Singapore, Brunei). The Philippines showed minimal change, with rates persistently above 940. Malaysia experienced a spike around 2000, followed by a decline, but then remained at a high level. Cambodia and Indonesia declined slowly (Figure 2B, Supplementary Table 5).

### Frontier analysis results for age‑standardized DALY rates of periodontal disease and edentulism, 1990 to 2023, based on SDI

**Fig. 3:**
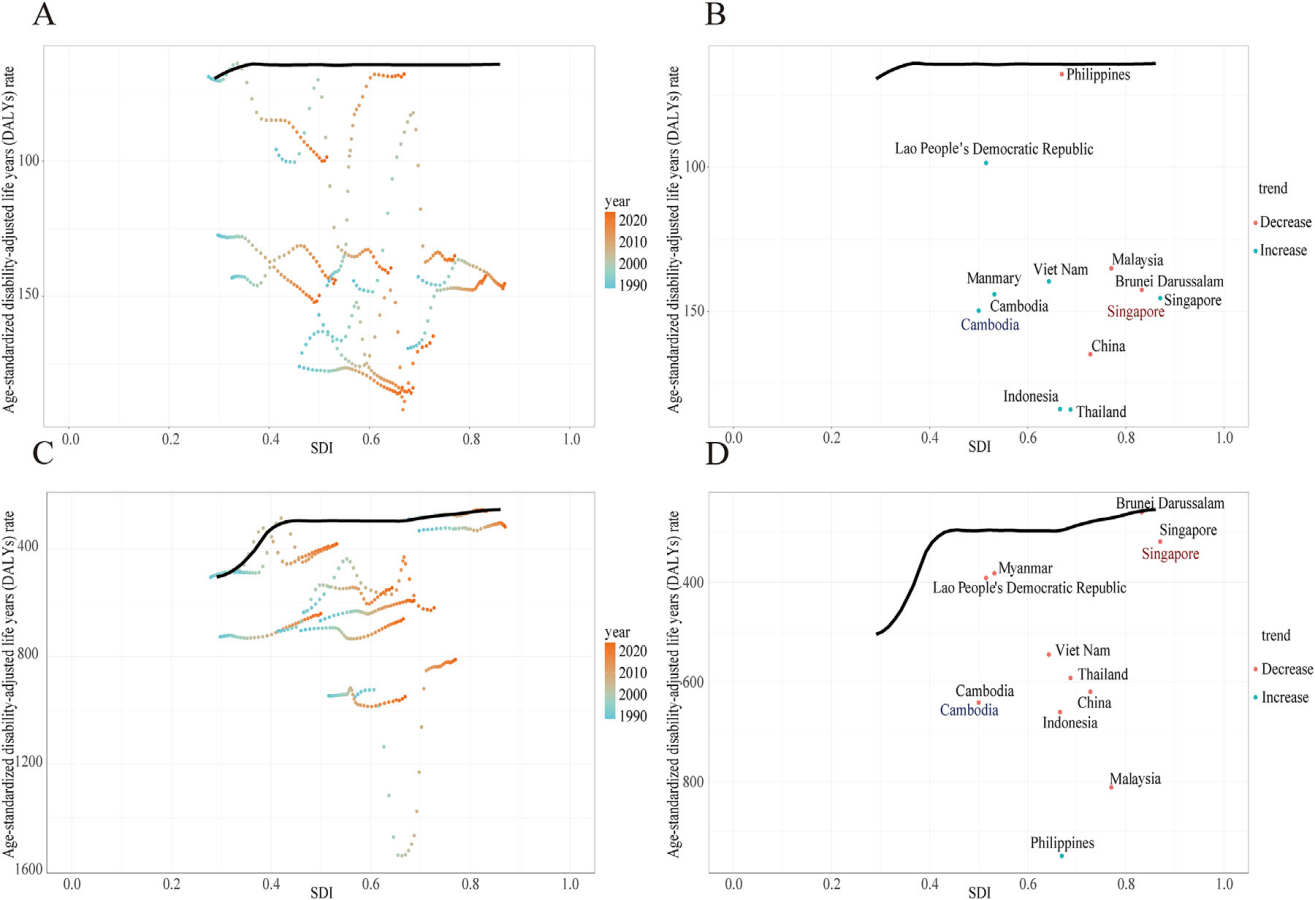
Frontier analysis of age-standardized disability-adjusted life years (DALYs) rates of periodontal disease (A and B) and edentulism (C and D) in China and ASEAN countries.

For periodontal disease, the frontier of age-standardized DALY rates showed a gently increasing trend across the entire SDI range, without steep changes. For edentulism, the frontier exhibited a typical S-shaped curve. In the low SDI range (below 0.4), the frontier rose rapidly and steeply, indicating that the baseline burden of edentulism increased markedly with increasing SDI at lower developmental levels. In the middle SDI range (0.4-0.6), the rate of increase slowed down. In the high SDI range (> 0.6), the frontier plateaus. The countries with the largest actual difference in potential improvement for both conditions were Lao PDR, Myanmar, the Philippines, Vietnam, Cambodia, Malaysia, Brunei Darussalam, Singapore, China, Indonesia, and Thailand. Cambodia has a lower SDI. Countries with a high SDI and high development potential, such as Singapore, showed a relatively high potential for improvement (Figure 3).

### ARIMA forecast results for age‑standardized DALY rates of periodontal disease and edentulism, 2024 to 2033

**Fig. 4:**
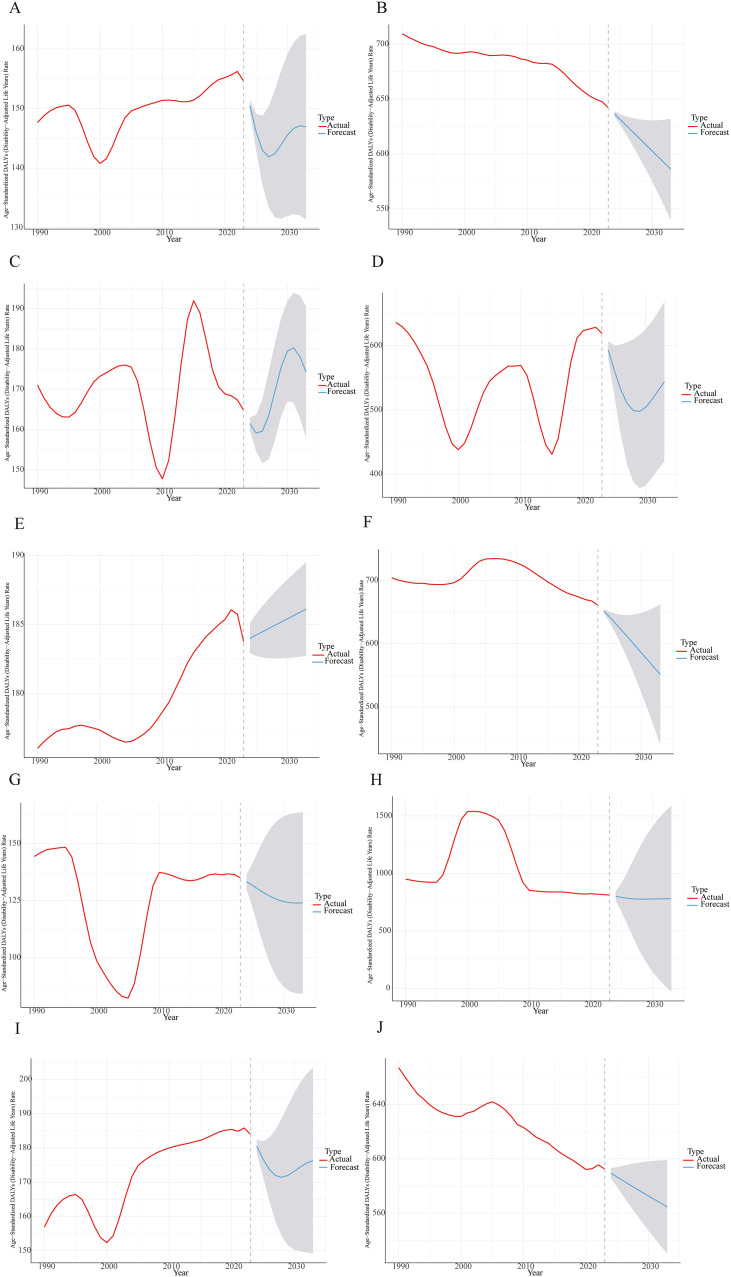
ARIMA analysis of age-standardized DALY rates for periodontal disease and edentulism in China and selected ASEAN countries. (A) Periodontal disease in ASEAN; (B) edentulism in ASEAN; (C) periodontal disease in China; (D) edentulism in China; (E) periodontal disease in Indonesia; (F) edentulism in Indonesia; (G) periodontal disease in Malaysia; (H) edentulism in Malaysia; (I) periodontal disease in Thailand; (J) edentulism in Thailand.

For periodontal disease (Figure 4A, C, E, G, I; Supplementary Tables 6, 8, 10, 12, 14): the ASEAN aggregate rate is forecasted to decrease from 150.48 (95% UI: 131.73-153.10) in 2024 to 146.93 (133.46-150.30) in 2033, with slight fluctuation around 2029-2031. China’s rate is projected to increase from 161.44 (157.31-181.38) in 2024, rising through 2033 and peaking near 2031. Indonesia shows a steady upward trend from 184.00 (182.55-187.33) in 2024 to 186.11 (182.57-186.84) in 2033. Malaysia continues its long‑term decline from 133.27 (95.72-157.31) in 2024 to 124.01 (102.94-153.07) in 2033. Thailand decreases from 180.50 (154.24-188.62) in 2024 to 176.31 (158.58-185.30) in 2033, slowing after 2030.

For edentulism (Figure 4B, D, F, H, J; Supplementary Tables 7, 9, 11, 13, 15): the ASEAN aggregate rate declines steadily from 636.37 in 2024 to 586.03 in 2033. China shows a clear decrease from 593.61 (388.41-609.90) in 2024 to 543.76 (416.37-605.01) in 2033, accelerating after 2025. Indonesia falls from 651.29 in 2024 to 551.19 in 2033. Malaysia declines from 801.33 (313.99-1238.24) in 2024 to 780.17 (423.13-1132.85) in 2033, remaining high but stable. Thailand drops gradually from 589.46 in 2024 to 564.63 in 2033, indicating slow but consistent improvement.

## Discussion

From 1990 to 2023, the burden of periodontal disease among older women increased significantly in Indonesia and Thailand, remained relatively stable in China, and declined in Malaysia. By contrast, the burden of edentulism showed a consistent and significant downward trend across most ASEAN regions. The Philippines and Malaysia sustained the highest edentulism burdens throughout the study period. Frontier analysis revealed that reducing edentulism is an achievable goal for socioeconomic development, as the frontier plateaus at higher SDI levels. For periodontal disease, the frontier increased only slightly, indicating that the baseline level of burden persists even in high-resource settings. The ARIMA projections suggest that edentulism will continue to decline through 2033 across the region, with the ASEAN aggregate forecast falling from approximately 636 to 586 DALYs per 100,000. In contrast, the incidence of periodontal disease is projected to increase further in China and Indonesia. These findings highlight the shifting and diverging nature of oral health challenges in the aging female population.

Co-occurrence analysis revealed three distinct patterns of disease burden across the study region. The dominant periodontal disease pattern was observed in Indonesia, Singapore, Myanmar, Brunei, and Thailand. In these countries, the DALY rate for periodontal disease exceeds that for edentulism. This pattern may reflect improved tooth retention in old age, leaving a larger dentate population susceptible to chronic periodontal inflammation.^16^ Greater access to restorative care allows individuals to retain more teeth; however, periodontal maintenance may not be adequately addressed. An edentulism-dominant pattern was identified in Malaysia, Lao PDR, and the Philippines. The Philippines presented the most extreme example, with the lowest recorded periodontal burden, but the highest edentulism burden in the region. However, this striking discordance requires careful interpretation. The GBD estimate for periodontal disease in the Philippines may underestimate the true burden owing to sparse primary data and a narrow case definition of severe periodontitis. A recent cross-sectional study of older Filipino adults found that 97.3 percent had periodontitis and 33.3 percent had severe periodontal destruction.^17^ This suggests that periodontal disease is far more prevalent than GBD estimates. In addition, the Philippines has an extraordinarily high burden of untreated dental caries, affecting approximately 92.4 percent of the population. Many older Filipinos may have experienced limited access to restorative care due to economic barriers and the geographic concentration of dentists in urban areas. Dental visits are often perceived as necessary when pain occurs. As time care is sought, extraction may be the only viable option for extensively damaged teeth.^18^ A consistent pattern, in which the burden levels for both conditions were similar, was observed in Cambodia, Vietnam, and China. These co-occurrence patterns underscore the importance of tailoring oral health strategies to the epidemiological profile of each country.^19^

Temporal analysis from 1990 to 2023 showed a slight but significant increase in the periodontal disease burden for the ASEAN aggregate. This increase was mainly driven by Thailand and Indonesia. This upward trend among older women may seem counterintuitive given the global improvements in oral hygiene awareness.^7^ This likely reflects the fact that fewer older adults become completely edentulous. As tooth retention increases, a larger pool of dentate individuals remain susceptible to age-related attachment loss and systemic conditions that worsen periodontal health. The phenomenon of more teeth being at risk has been documented in aging populations worldwide.^20^ The 2023 data points also showed marked heterogeneity in the age-standardized DALY rates. Thailand and Indonesia have the highest periodontal disease burden, exceeding 180 per 100,000 people. The high burden in these countries may be explained in part by differences in the prevalence of risk factors such as smoking and diabetes.^21^ Indonesia has a very high rate of male smoking, and a high prevalence of diabetes also likely contributes to the periodontal burden seen in older Indonesian women.^22^ For edentulism, the ASEAN regional rate has decreased from 709.39 in 1990 to 641.96 in 2023. Most countries, including China, Thailand, Vietnam, Lao PDR, Myanmar, Singapore, and Brunei, showed a decreasing trend. This decline is a notable public health achievement and reflects decades of improvement in oral hygiene, fluoride use, and a shift towards tooth preservation.^7^ The Philippines showed minimal change, with rates persistently above 940 per 100,000. Malaysia experienced a spike around 2000, followed by a decline, but then remained at a high level.

Frontier analysis provided insights into the relationship between socioeconomic development and oral diseases. The frontier for periodontal disease increased only slightly across the full SDI range, indicating that the baseline burden persists even in high‑resource settings. This is likely because periodontitis is a complex, multifactorial disease driven not only by socio‑economic factors but also by genetic predisposition, smoking, diabetes, and age‑related immunological changes. Unlike edentulism, which can be nearly eliminated through comprehensive preventive care and restorative services, periodontal disease is more refractory to purely economic advancement. This contrasts sharply with the S‑shaped frontier for edentulism, where higher SDI levels are associated with a clear plateau, suggesting that tooth loss is largely preventable with adequate resources.^23^^,^^24^ By contrast, the frontier for edentulism showed a steep increase at low SDI levels and then plateaued at higher SDI levels. At low development levels, even small improvements in access may lead to more recorded edentulism, as people finally seek care for long-neglected dental conditions.^25^ As development progresses, the plateau indicates that a low burden of edentulism is achievable with robust preventive care. The substantial gap between observed rates and the frontier for countries such as the Philippines and Cambodia underscores the major potential for health improvement. Closing this gap requires targeted policy intervention. These include integrating oral health into primary care, expanding dental coverage for older adults, and promoting the use of fluoride and pit and fissure sealants from an early age.^26^

The ARIMA projections for 2033 suggest that edentulism rates will continue to decline across most regions. For the ASEAN aggregate, the edentulism DALY rate is forecasted to decrease from 636.37 in 2024 to 586.03 in 2033. China is projected to decline from 593.61 to 543.76, and Indonesia from 651.29 to 551.19 over the same period. Malaysia and Thailand are also expected to exhibit a gradual decrease.^27^ The projected increase in China and Indonesia may reflect rapidly aging populations with increasing tooth retention, alongside persistently high prevalence of risk factors such as smoking (in Indonesia) and diabetes (in both countries). In contrast, the projected decline in Malaysia and Thailand could be attributable to more established oral health promotion programmes, greater public awareness, and better primary care integration, although these hypotheses require further investigation. These projected declines are encouraging, and suggest that current public health efforts are yielding sustained benefits. In contrast, periodontal disease burden is projected to follow a more varied path. The ASEAN aggregate and Indonesia are forecast to see continued increases in periodontal DALY rates. China is projected to see an increase by 2033.^28^ These projections are based on historical trends and serve as warning. Without accelerated action, the burden of periodontal disease among older women will not decrease. It may intensify as the population ages and retains more teeth.^29^ The projected rise in periodontal burden calls for a strategic shift in the oral health policy. Emphasis must move beyond tooth retention to include long-term management of periodontal health.^30^ This includes integrating periodontal screening into routine primary care for older adults, ensuring reimbursement for nonsurgical periodontal therapy, and raising awareness of the links between periodontal disease and systemic conditions.

This study has several limitations. First, GBD definitions focus on severe periodontitis, likely underestimating milder cases. In particular, GBD’s narrow definition may substantially underestimate the true burden in countries like the Philippines, where a recent survey reported that 97.3% of older adults had some form of periodontitis. This discrepancy underscores the need for caution when interpreting GBD estimates for countries with limited primary data, and we have now highlighted this in the limitations. Furthermore, the accuracy of GBD estimates is contingent on the availability and quality of primary surveillance data in each country; for nations with sparse epidemiological infrastructure, the modelled estimates may carry greater uncertainty. Additionally, while GBD provides the most comprehensive global estimates, our results have not been independently validated against local epidemiological surveys in each country. Future studies should compare GBD trends with national surveillance data to improve accuracy. Second, we could not include behavioural, economic, or healthcare access variables due to data constraints. Third, ARIMA forecasts are based solely on historical patterns (1990-2023) and should be viewed as trend-based illustrations rather than deterministic predictions. Moreover, the COVID-19 pandemic (2020-2021) led to widespread suspension of routine dental services and screening, which may have temporarily underestimated mild periodontal cases while also delaying care for progressive disease. Postpandemic catch-up visits could have caused artificial rebounds in diagnosed cases. Such fluctuations may introduce bias in both historical trends and ARIMA forecasts. Our projections should therefore be interpreted with particular caution, especially for the post-2020 period

## Conclusion

Based on GBD 2023 data, the oral health burden for older women in China and ASEAN countries shifted from 1990 to 2023: edentulism declined steadily, but periodontal disease rose in Indonesia, Thailand, and China. This highlights the transition from tooth loss to chronic inflammatory management in aging populations. Country‑specific strategies are urgently needed: for high‑burden periodontal countries (Indonesia, Thailand, China), investments in periodontal screening and non‑surgical therapy should be prioritised; for high‑edentulism countries (Philippines, Malaysia), expanding restorative and preventive dental coverage for older women is critical.

## Ethics statement and consent to participate

Ethical approval was not required for this study, as it relied exclusively on publicly available, anonymized data from the Global Burden of Disease Study 2023.

## Declaration of generative AI and AI-assisted technologies in the writing process

During the preparation of this work, the authors did not use any generative AI- or AI-assisted technologies for data analysis, interpretation, or manuscript writing. Grammarly Business was solely used for grammar and spelling checks. The authors take full responsibility for the content and integrity of this study.

## Data availability

All data used in this study are publicly available from the Global Burden of Disease 2023 repository (https://ghdx.healthdata.org/gbd-2023).

## Funding

This study was supported by the Shandong Provincial Natural Science Foundation (grant no. ZR2025QC908), and National Traditional Chinese Medicine Comprehensive Reform Demonstration Zone Science and Technology Co-Construction Project (Grant No. GZY-KJS-SD-2024-106). The funders had no role in the study design, data collection, analysis, interpretation, manuscript writing, or decision to submit for publication.

## Author contributions

Yilin Zhang: Conceptualization, data curation, formal analysis, writing – original draft, visualization. Xiaowei Dai: Methodology, software, validation, writing – review and editing, visualization. Yuan Tang: Data curation, formal analysis, investigation, writing – original draft, project administration. Jing Li: Validation, formal analysis, resources, writing – review and editing. Fei He: Conceptualization, funding acquisition, supervision, writing – review and editing. Lei Zhao: Methodology, supervision, funding acquisition, writing – review and editing. Xin Xu: Conceptualization, project administration, supervision, funding acquisition, writing – review and editing. All authors read and approved the final manuscript.

## Conflict of interest

The authors declare that they have no known competing financial interests or personal relationships that could have appeared to influence the work reported in this article.

## Appendix Supplementary materials

Image, application 1

## Footnotes

Supplementary material associated with this article can be found in the online version at doi:10.1016/j.identj.2026.109780.
