# Supplementary material for: Periodontal Disease and Edentulism Trends in Older Women of China and Association of Southeast Asian Nations, 1990 to 2033
Source: Int Dent J. 2026 Jul 24;76(5):109780. doi: 10.1016/j.identj.2026.109780 (PMC13425883; doi:10.1016/j.identj.2026.109780)
Supplement: Supplementary file 1 [file mmc1.pdf]

Supplementary Materials

Supplemental Table S1: Spatial Distribution Map of Periodontal Diseases in 2023 1
Supplemental Table S2: Spatial Distribution Map of Edentulism in 2023 2
Supplemental Table S3: Comorbidity Spatial Distribution Map of Periodontal Diseases and Edentulism in 2023 3
Supplemental Table S4: Trend Analysis of Periodontal Diseases from 1990 to 2023 4
Supplemental Table S5: Trend Analysis of Edentulism from 1990 to 2023 25
Supplemental Table S6: Trend Projection of Periodontal Diseases from 2023 to 2033 - ASEAN 46
Supplemental Table S7: Trend Projection of Edentulism from 2023 to 2033 - ASEAN 48
Supplemental Table S8: Trend Projection of Periodontal Diseases from 2023 to 2033 - China 50
Supplemental Table S9: Trend Projection of Edentulism from 2023 to 2033 - China 52
Supplemental Table S10: Trend Projection of Periodontal Diseases from 2023 to 2033 - Indonesia 54
Supplemental Table S11: Trend Projection of Edentulism from 2023 to 2033 - Indonesia 56
Supplemental Table S12: Trend Projection of Periodontal Diseases from 2023 to 2033 - Malaysia 58
Supplemental Table S13: Trend Projection of Edentulism from 2023 to 2033 - Malaysia 60
Supplemental Table S14: Trend Projection of Periodontal Diseases from 2023 to 2033 - Thailand 62
Supplemental Table S15: Trend Projection of Edentulism from 2023 to 2033 - Thailand 64

Supplemental Table S1: Spatial Distribution Map of Periodontal Diseases in 2023

	location_id	location	disease1	val
1	11	Indonesia	183.77	183.77
2	13	Malaysia	135.03	135.03
3	10	Cambodia	149.71	149.71
4	12	Lao People's Democratic Republic	98.59	98.59
5	20	Viet Nam	139.54	139.54
6	69	Singapore	145.35	145.35
7	15	Myanmar	144.00	144.00
8	6	China	164.78	164.78
9	66	Brunei Darussalam	142.45	142.45
10	16	Philippines	67.85	67.85
11	18	Thailand	183.93	183.93

Supplemental Table S2: Spatial Distribution Map of Edentulism in 2023

	location_id	location	disease2	val
1	11	Indonesia	660.54	660.54
2	13	Malaysia	811.45	811.45
3	10	Cambodia	641.18	641.18
4	12	Lao People's Democratic Republic	391.55	391.55
5	20	Viet Nam	545.32	545.32
6	69	Singapore	318.65	318.65
7	15	Myanmar	382.28	382.28
8	6	China	619.35	619.35
9	66	Brunei Darussalam	259.81	259.81
10	16	Philippines	948.96	948.96
11	18	Thailand	592.45	592.45

Supplemental Table S3: Comorbidity Spatial Distribution Map of Periodontal Diseases and Edentulism in 2023

	location_id	comparison	location
1	11	Periodontal diseases dominant	Indonesia
2	13	Edentulism dominant	Malaysia
3	10	Consistent	Cambodia
4	12	Edentulism dominant	Lao People's Democratic Republic
5	20	Consistent	Viet Nam
6	69	Periodontal diseases dominant	Singapore
7	15	Periodontal diseases dominant	Myanmar
8	6	Consistent	China
9	66	Periodontal diseases dominant	Brunei Darussalam
10	16	Edentulism dominant	Philippines
11	18	Periodontal diseases dominant	Thailand

Supplemental Table S4: Trend Analysis of Periodontal Diseases from 1990 to 2023

	population_group_name	measure_name	location_name	sex_name	age_name	cause_name	metric_name	year	val	upper	lower
1	All Population	DALYs (Disability-Adjusted Life Years)	Cambodia	Female	60+ years	Periodontal diseases	Rate	1991	127.69	284.57	47.61
2	All Population	DALYs (Disability-Adjusted Life Years)	Philippines	Female	60+ years	Periodontal diseases	Rate	1990	146.01	309.62	54.67
3	All Population	DALYs (Disability-Adjusted Life Years)	Thailand	Female	60+ years	Periodontal diseases	Rate	1990	156.89	337.33	60.61
4	All Population	DALYs (Disability-Adjusted Life Years)	Myanmar	Female	60+ years	Periodontal diseases	Rate	1990	143.22	307.88	53.06
5	All Population	DALYs (Disability-Adjusted Life Years)	Myanmar	Female	60+ years	Periodontal diseases	Rate	1991	142.93	307.96	52.89
6	All Population	DALYs (Disability-Adjusted Life Years)	Malaysia	Female	60+ years	Periodontal diseases	Rate	1991	146.01	308.20	53.89
7	All Population	DALYs (Disability-Adjusted Life Years)	China	Female	60+ years	Periodontal diseases	Rate	1990	170.99	351.00	65.17
8	All Population	DALYs (Disability-Adjusted Life Years)	Brunei Darussalam	Female	60+ years	Periodontal diseases	Rate	1990	169.33	357.74	64.52
9	All Population	DALYs (Disability-Adjusted Life Years)	Indonesia	Female	60+ years	Periodontal diseases	Rate	1991	176.50	366.80	67.49
10	All Population	DALYs (Disability-Adjusted Life Years)	Singapore	Female	60+ years	Periodontal diseases	Rate	1991	144.19	309.36	54.56
11	All Population	DALYs (Disability-Adjusted Life Years)	Viet Nam	Female	60+ years	Periodontal diseases	Rate	1991	97.93	219.34	36.91
12	All Population	DALYs (Disability-Adjusted Life Years)	China	Female	60+ years	Periodontal diseases	Rate	1991	167.88	346.09	63.76
13	All Population	DALYs (Disability-Adjusted Life Years)	Myanmar	Female	60+ years	Periodontal diseases	Rate	1992	142.74	304.99	52.97
14	All Population	DALYs (Disability-Adjusted Life Years)	Cambodia	Female	60+ years	Periodontal diseases	Rate	1990	127.40	282.98	47.31
15	All Population	DALYs (Disability-Adjusted Life Years)	Lao People's Democratic Republic	Female	60+ years	Periodontal diseases	Rate	1992	69.84	153.88	24.65
16	All Population	DALYs (Disability-Adjusted Life Years)	Singapore	Female	60+ years	Periodontal diseases	Rate	1990	142.81	306.76	53.37
17	All Population	DALYs (Disability-Adjusted Life Years)	Association of Southeast Asian Nations	Female	60+ years	Periodontal diseases	Rate	1991	148.77	313.83	56.45
18	All Population	DALYs (Disability-Adjusted Life Years)	Viet Nam	Female	60+ years	Periodontal diseases	Rate	1990	95.73	215.98	35.96
19	All Population	DALYs (Disability-Adjusted Life Years)	Lao People's Democratic Republic	Female	60+ years	Periodontal diseases	Rate	1991	69.38	150.87	24.10
20	All Population	DALYs (Disability-Adjusted Life Years)	Philippines	Female	60+ years	Periodontal	Rate	1991	144.40	305.95	54.23

						diseases					
21	All Population	DALYs (Disability-Adjusted Life Years)	Thailand	Female	60+ years	Periodontal diseases	Rate	1991	160.60	343.21	61.29
22	All Population	DALYs (Disability-Adjusted Life Years)	Indonesia	Female	60+ years	Periodontal diseases	Rate	1993	177.24	363.78	67.92
23	All Population	DALYs (Disability-Adjusted Life Years)	Indonesia	Female	60+ years	Periodontal diseases	Rate	1990	176.03	366.67	67.48
24	All Population	DALYs (Disability-Adjusted Life Years)	Association of Southeast Asian Nations	Female	60+ years	Periodontal diseases	Rate	1990	147.65	311.06	56.11
25	All Population	DALYs (Disability-Adjusted Life Years)	Brunei Darussalam	Female	60+ years	Periodontal diseases	Rate	1991	169.04	355.39	64.21
26	All Population	DALYs (Disability-Adjusted Life Years)	China	Female	60+ years	Periodontal diseases	Rate	1992	165.54	343.44	62.88
27	All Population	DALYs (Disability-Adjusted Life Years)	Myanmar	Female	60+ years	Periodontal diseases	Rate	1993	142.62	305.98	52.68
28	All Population	DALYs (Disability-Adjusted Life Years)	Singapore	Female	60+ years	Periodontal diseases	Rate	1993	145.58	313.36	55.83
29	All Population	DALYs (Disability-Adjusted Life Years)	Singapore	Female	60+ years	Periodontal diseases	Rate	1994	145.79	311.79	56.01
30	All Population	DALYs (Disability-Adjusted Life Years)	Singapore	Female	60+ years	Periodontal diseases	Rate	1992	145.03	309.50	55.97
31	All Population	DALYs (Disability-Adjusted Life Years)	China	Female	60+ years	Periodontal diseases	Rate	1993	163.97	341.10	62.04
32	All Population	DALYs (Disability-Adjusted Life Years)	Philippines	Female	60+ years	Periodontal diseases	Rate	1992	142.92	302.72	53.66
33	All Population	DALYs (Disability-Adjusted Life Years)	Brunei Darussalam	Female	60+ years	Periodontal diseases	Rate	1993	168.22	358.34	63.54
34	All Population	DALYs (Disability-Adjusted Life Years)	Philippines	Female	60+ years	Periodontal diseases	Rate	1993	141.83	300.56	53.36
35	All Population	DALYs (Disability-Adjusted Life Years)	Association of Southeast Asian Nations	Female	60+ years	Periodontal diseases	Rate	1992	149.59	315.17	56.63
36	All Population	DALYs (Disability-Adjusted Life Years)	Indonesia	Female	60+ years	Periodontal diseases	Rate	1994	177.41	363.33	68.20
37	All Population	DALYs (Disability-Adjusted Life Years)	Cambodia	Female	60+ years	Periodontal diseases	Rate	1994	128.29	286.93	47.09
38	All Population	DALYs (Disability-Adjusted Life Years)	Cambodia	Female	60+ years	Periodontal diseases	Rate	1992	127.98	285.48	47.82
39	All Population	DALYs (Disability-Adjusted Life Years)	Cambodia	Female	60+ years	Periodontal diseases	Rate	1993	128.09	286.77	47.25
40	All Population	DALYs (Disability-Adjusted Life Years)	Cambodia	Female	60+ years	Periodontal diseases	Rate	1995	128.20	288.89	47.01
41	All Population	DALYs (Disability-Adjusted Life Years)	Brunei	Female	60+ years	Periodontal	Rate	1992	168.70	360.58	63.61

			Darussalam			diseases					
42	All Population	DALYs (Disability-Adjusted Life Years)	Viet Nam	Female	60+ years	Periodontal diseases	Rate	1994	100.31	220.49	36.64
43	All Population	DALYs (Disability-Adjusted Life Years)	Lao People's Democratic Republic	Female	60+ years	Periodontal diseases	Rate	1990	68.79	147.07	23.75
44	All Population	DALYs (Disability-Adjusted Life Years)	Indonesia	Female	60+ years	Periodontal diseases	Rate	1992	176.90	366.51	67.59
45	All Population	DALYs (Disability-Adjusted Life Years)	Cambodia	Female	60+ years	Periodontal diseases	Rate	1996	128.14	286.72	47.38
46	All Population	DALYs (Disability-Adjusted Life Years)	Indonesia	Female	60+ years	Periodontal diseases	Rate	1995	177.45	362.41	68.06
47	All Population	DALYs (Disability-Adjusted Life Years)	Cambodia	Female	60+ years	Periodontal diseases	Rate	1997	128.16	281.83	47.70
48	All Population	DALYs (Disability-Adjusted Life Years)	Myanmar	Female	60+ years	Periodontal diseases	Rate	1995	142.73	306.27	53.12
49	All Population	DALYs (Disability-Adjusted Life Years)	Malaysia	Female	60+ years	Periodontal diseases	Rate	1993	147.77	313.31	54.94
50	All Population	DALYs (Disability-Adjusted Life Years)	Lao People's Democratic Republic	Female	60+ years	Periodontal diseases	Rate	1994	70.31	158.64	25.35
51	All Population	DALYs (Disability-Adjusted Life Years)	Malaysia	Female	60+ years	Periodontal diseases	Rate	1990	144.35	305.58	53.70
52	All Population	DALYs (Disability-Adjusted Life Years)	Thailand	Female	60+ years	Periodontal diseases	Rate	1992	163.23	349.79	62.17
53	All Population	DALYs (Disability-Adjusted Life Years)	Brunei Darussalam	Female	60+ years	Periodontal diseases	Rate	1994	167.33	357.14	63.07
54	All Population	DALYs (Disability-Adjusted Life Years)	Thailand	Female	60+ years	Periodontal diseases	Rate	1993	165.00	353.00	62.66
55	All Population	DALYs (Disability-Adjusted Life Years)	Myanmar	Female	60+ years	Periodontal diseases	Rate	1994	142.64	306.19	53.50
56	All Population	DALYs (Disability-Adjusted Life Years)	Lao People's Democratic Republic	Female	60+ years	Periodontal diseases	Rate	1993	70.09	155.62	24.99
57	All Population	DALYs (Disability-Adjusted Life Years)	Cambodia	Female	60+ years	Periodontal diseases	Rate	1999	128.03	280.10	48.24
58	All Population	DALYs (Disability-Adjusted Life Years)	Thailand	Female	60+ years	Periodontal diseases	Rate	1995	166.41	353.58	63.68
59	All Population	DALYs (Disability-Adjusted Life Years)	Lao People's Democratic Republic	Female	60+ years	Periodontal diseases	Rate	1995	70.37	159.94	25.70
60	All Population	DALYs (Disability-Adjusted Life Years)	Philippines	Female	60+ years	Periodontal diseases	Rate	1994	141.11	297.77	53.09
61	All Population	DALYs (Disability-Adjusted Life Years)	Singapore	Female	60+ years	Periodontal	Rate	1996	144.93	292.96	54.26

						diseases					
62	All Population	DALYs (Disability-Adjusted Life Years)	Viet Nam	Female	60+ years	Periodontal diseases	Rate	1996	97.29	208.99	36.55
63	All Population	DALYs (Disability-Adjusted Life Years)	Lao People's Democratic Republic	Female	60+ years	Periodontal diseases	Rate	1996	69.78	156.29	25.19
64	All Population	DALYs (Disability-Adjusted Life Years)	China	Female	60+ years	Periodontal diseases	Rate	1995	163.08	341.61	61.46
65	All Population	DALYs (Disability-Adjusted Life Years)	Singapore	Female	60+ years	Periodontal diseases	Rate	1995	145.93	315.38	55.87
66	All Population	DALYs (Disability-Adjusted Life Years)	Malaysia	Female	60+ years	Periodontal diseases	Rate	1994	148.14	312.16	54.77
67	All Population	DALYs (Disability-Adjusted Life Years)	Philippines	Female	60+ years	Periodontal diseases	Rate	1996	140.31	295.75	52.83
68	All Population	DALYs (Disability-Adjusted Life Years)	Thailand	Female	60+ years	Periodontal diseases	Rate	1996	164.97	349.37	63.34
69	All Population	DALYs (Disability-Adjusted Life Years)	Association of Southeast Asian Nations	Female	60+ years	Periodontal diseases	Rate	1993	150.13	314.58	56.69
70	All Population	DALYs (Disability-Adjusted Life Years)	Viet Nam	Female	60+ years	Periodontal diseases	Rate	1995	100.44	221.79	36.65
71	All Population	DALYs (Disability-Adjusted Life Years)	China	Female	60+ years	Periodontal diseases	Rate	1994	163.15	341.38	61.55
72	All Population	DALYs (Disability-Adjusted Life Years)	Lao People's Democratic Republic	Female	60+ years	Periodontal diseases	Rate	2000	63.83	134.54	23.96
73	All Population	DALYs (Disability-Adjusted Life Years)	Thailand	Female	60+ years	Periodontal diseases	Rate	1994	166.01	355.55	63.34
74	All Population	DALYs (Disability-Adjusted Life Years)	Association of Southeast Asian Nations	Female	60+ years	Periodontal diseases	Rate	1994	150.39	314.83	56.86
75	All Population	DALYs (Disability-Adjusted Life Years)	Cambodia	Female	60+ years	Periodontal diseases	Rate	2000	128.00	275.05	48.56
76	All Population	DALYs (Disability-Adjusted Life Years)	China	Female	60+ years	Periodontal diseases	Rate	1997	166.59	351.11	63.02
77	All Population	DALYs (Disability-Adjusted Life Years)	Philippines	Female	60+ years	Periodontal diseases	Rate	1998	137.05	287.85	51.45
78	All Population	DALYs (Disability-Adjusted Life Years)	Lao People's Democratic Republic	Female	60+ years	Periodontal diseases	Rate	1997	68.07	148.48	25.00
79	All Population	DALYs (Disability-Adjusted Life Years)	Philippines	Female	60+ years	Periodontal diseases	Rate	1995	140.90	297.21	53.17
80	All Population	DALYs (Disability-Adjusted Life Years)	Indonesia	Female	60+ years	Periodontal diseases	Rate	1997	177.71	363.75	67.70

81	All Population	DALYs (Disability-Adjusted Life Years)	Philippines	Female	60+ years	Periodontal diseases	Rate	1997	138.84	292.10	52.14
82	All Population	DALYs (Disability-Adjusted Life Years)	Cambodia	Female	60+ years	Periodontal diseases	Rate	1998	127.94	276.59	47.57
83	All Population	DALYs (Disability-Adjusted Life Years)	Cambodia	Female	60+ years	Periodontal diseases	Rate	2001	128.59	280.24	48.91
84	All Population	DALYs (Disability-Adjusted Life Years)	Lao People's Democratic Republic	Female	60+ years	Periodontal diseases	Rate	1999	64.47	135.78	23.72
85	All Population	DALYs (Disability-Adjusted Life Years)	Cambodia	Female	60+ years	Periodontal diseases	Rate	2002	129.68	280.57	49.41
86	All Population	DALYs (Disability-Adjusted Life Years)	Philippines	Female	60+ years	Periodontal diseases	Rate	2000	134.69	282.94	50.53
87	All Population	DALYs (Disability-Adjusted Life Years)	Malaysia	Female	60+ years	Periodontal diseases	Rate	1995	148.39	311.70	54.66
88	All Population	DALYs (Disability-Adjusted Life Years)	Viet Nam	Female	60+ years	Periodontal diseases	Rate	1993	100.15	220.24	37.23
89	All Population	DALYs (Disability-Adjusted Life Years)	Malaysia	Female	60+ years	Periodontal diseases	Rate	1992	147.35	311.66	54.79
90	All Population	DALYs (Disability-Adjusted Life Years)	Singapore	Female	60+ years	Periodontal diseases	Rate	1999	137.61	271.12	49.10
91	All Population	DALYs (Disability-Adjusted Life Years)	Brunei Darussalam	Female	60+ years	Periodontal diseases	Rate	1999	150.10	304.95	52.17
92	All Population	DALYs (Disability-Adjusted Life Years)	Brunei Darussalam	Female	60+ years	Periodontal diseases	Rate	1998	154.29	315.61	54.36
93	All Population	DALYs (Disability-Adjusted Life Years)	Myanmar	Female	60+ years	Periodontal diseases	Rate	2000	146.06	312.86	55.78
94	All Population	DALYs (Disability-Adjusted Life Years)	Indonesia	Female	60+ years	Periodontal diseases	Rate	1998	177.61	364.02	67.62
95	All Population	DALYs (Disability-Adjusted Life Years)	Indonesia	Female	60+ years	Periodontal diseases	Rate	1996	177.64	363.02	67.98
96	All Population	DALYs (Disability-Adjusted Life Years)	Cambodia	Female	60+ years	Periodontal diseases	Rate	2004	132.74	281.74	51.19
97	All Population	DALYs (Disability-Adjusted Life Years)	Lao People's Democratic Republic	Female	60+ years	Periodontal diseases	Rate	1998	66.11	143.86	24.10
98	All Population	DALYs (Disability-Adjusted Life Years)	Thailand	Female	60+ years	Periodontal diseases	Rate	1998	157.16	336.20	59.39
99	All Population	DALYs (Disability-Adjusted Life Years)	Cambodia	Female	60+ years	Periodontal diseases	Rate	2005	134.07	283.61	50.87
100	All Population	DALYs (Disability-Adjusted Life Years)	Singapore	Female	60+ years	Periodontal diseases	Rate	2001	136.77	270.69	46.75
101	All Population	DALYs (Disability-Adjusted Life Years)	Indonesia	Female	60+ years	Periodontal diseases	Rate	2000	177.34	363.02	68.02

102	All Population	DALYs (Disability-Adjusted Life Years)	Malaysia	Female	60+ years	Periodontal diseases	Rate	1999	106.64	217.33	39.48
103	All Population	DALYs (Disability-Adjusted Life Years)	Philippines	Female	60+ years	Periodontal diseases	Rate	2001	130.82	271.88	50.36
104	All Population	DALYs (Disability-Adjusted Life Years)	Thailand	Female	60+ years	Periodontal diseases	Rate	1997	161.42	344.89	61.82
105	All Population	DALYs (Disability-Adjusted Life Years)	Philippines	Female	60+ years	Periodontal diseases	Rate	2002	121.82	251.62	47.53
106	All Population	DALYs (Disability-Adjusted Life Years)	Philippines	Female	60+ years	Periodontal diseases	Rate	1999	135.47	284.90	50.81
107	All Population	DALYs (Disability-Adjusted Life Years)	China	Female	60+ years	Periodontal diseases	Rate	1999	171.80	363.20	65.45
108	All Population	DALYs (Disability-Adjusted Life Years)	Thailand	Female	60+ years	Periodontal diseases	Rate	2000	152.37	326.28	57.88
109	All Population	DALYs (Disability-Adjusted Life Years)	Philippines	Female	60+ years	Periodontal diseases	Rate	2004	102.22	209.37	39.30
110	All Population	DALYs (Disability-Adjusted Life Years)	Association of Southeast Asian Nations	Female	60+ years	Periodontal diseases	Rate	1995	150.55	314.37	56.84
111	All Population	DALYs (Disability-Adjusted Life Years)	Thailand	Female	60+ years	Periodontal diseases	Rate	2004	171.57	335.72	63.93
112	All Population	DALYs (Disability-Adjusted Life Years)	Singapore	Female	60+ years	Periodontal diseases	Rate	2002	137.24	273.72	46.63
113	All Population	DALYs (Disability-Adjusted Life Years)	Lao People's Democratic Republic	Female	60+ years	Periodontal diseases	Rate	2001	65.93	138.03	24.63
114	All Population	DALYs (Disability-Adjusted Life Years)	Lao People's Democratic Republic	Female	60+ years	Periodontal diseases	Rate	2002	70.95	148.38	25.88
115	All Population	DALYs (Disability-Adjusted Life Years)	Philippines	Female	60+ years	Periodontal diseases	Rate	2005	98.17	199.83	37.17
116	All Population	DALYs (Disability-Adjusted Life Years)	Cambodia	Female	60+ years	Periodontal diseases	Rate	2006	135.66	286.18	51.70
117	All Population	DALYs (Disability-Adjusted Life Years)	Cambodia	Female	60+ years	Periodontal diseases	Rate	2007	137.27	289.42	51.98
118	All Population	DALYs (Disability-Adjusted Life Years)	Brunei Darussalam	Female	60+ years	Periodontal diseases	Rate	1995	166.14	356.30	63.16
119	All Population	DALYs (Disability-Adjusted Life Years)	Thailand	Female	60+ years	Periodontal diseases	Rate	2003	165.91	321.76	63.14
120	All Population	DALYs (Disability-Adjusted Life Years)	China	Female	60+ years	Periodontal diseases	Rate	2000	173.21	365.96	66.03
121	All Population	DALYs (Disability-Adjusted Life Years)	China	Female	60+ years	Periodontal diseases	Rate	1996	164.22	344.93	62.00
122	All Population	DALYs (Disability-Adjusted Life Years)	Brunei	Female	60+ years	Periodontal	Rate	1997	159.18	323.77	58.19

			Darussalam			diseases					
123	All Population	DALYs (Disability-Adjusted Life Years)	Myanmar	Female	60+ years	Periodontal diseases	Rate	1997	143.85	307.86	54.29
124	All Population	DALYs (Disability-Adjusted Life Years)	Lao People's Democratic Republic	Female	60+ years	Periodontal diseases	Rate	2004	81.99	177.21	29.21
125	All Population	DALYs (Disability-Adjusted Life Years)	Lao People's Democratic Republic	Female	60+ years	Periodontal diseases	Rate	2005	84.41	186.16	29.26
126	All Population	DALYs (Disability-Adjusted Life Years)	Myanmar	Female	60+ years	Periodontal diseases	Rate	2001	145.07	301.26	54.99
127	All Population	DALYs (Disability-Adjusted Life Years)	Brunei Darussalam	Female	60+ years	Periodontal diseases	Rate	2002	147.47	296.86	51.35
128	All Population	DALYs (Disability-Adjusted Life Years)	Myanmar	Female	60+ years	Periodontal diseases	Rate	2002	142.39	292.87	52.88
129	All Population	DALYs (Disability-Adjusted Life Years)	Thailand	Female	60+ years	Periodontal diseases	Rate	1999	153.76	329.11	58.56
130	All Population	DALYs (Disability-Adjusted Life Years)	Malaysia	Female	60+ years	Periodontal diseases	Rate	1997	133.07	275.66	49.18
131	All Population	DALYs (Disability-Adjusted Life Years)	Association of Southeast Asian Nations	Female	60+ years	Periodontal diseases	Rate	1998	144.38	303.54	54.85
132	All Population	DALYs (Disability-Adjusted Life Years)	Brunei Darussalam	Female	60+ years	Periodontal diseases	Rate	2001	147.76	298.85	50.95
133	All Population	DALYs (Disability-Adjusted Life Years)	Association of Southeast Asian Nations	Female	60+ years	Periodontal diseases	Rate	1999	141.91	297.12	54.03
134	All Population	DALYs (Disability-Adjusted Life Years)	Brunei Darussalam	Female	60+ years	Periodontal diseases	Rate	1996	163.62	331.44	61.46
135	All Population	DALYs (Disability-Adjusted Life Years)	Thailand	Female	60+ years	Periodontal diseases	Rate	2002	159.55	314.77	62.34
136	All Population	DALYs (Disability-Adjusted Life Years)	Indonesia	Female	60+ years	Periodontal diseases	Rate	2002	176.84	363.95	67.48
137	All Population	DALYs (Disability-Adjusted Life Years)	Viet Nam	Female	60+ years	Periodontal diseases	Rate	1992	99.34	219.20	37.31
138	All Population	DALYs (Disability-Adjusted Life Years)	Philippines	Female	60+ years	Periodontal diseases	Rate	2003	111.19	228.78	43.25
139	All Population	DALYs (Disability-Adjusted Life Years)	Thailand	Female	60+ years	Periodontal diseases	Rate	2001	154.34	315.39	59.08
140	All Population	DALYs (Disability-Adjusted Life Years)	Lao People's Democratic Republic	Female	60+ years	Periodontal diseases	Rate	2006	84.90	188.77	29.56
141	All Population	DALYs (Disability-Adjusted Life Years)	Cambodia	Female	60+ years	Periodontal diseases	Rate	2008	139.22	294.58	51.59

142	All Population	DALYs (Disability-Adjusted Life Years)	Lao People's Democratic Republic	Female	60+ years	Periodontal diseases	Rate	2008	84.91	183.93	30.52
143	All Population	DALYs (Disability-Adjusted Life Years)	China	Female	60+ years	Periodontal diseases	Rate	2001	174.06	365.58	66.51
144	All Population	DALYs (Disability-Adjusted Life Years)	Thailand	Female	60+ years	Periodontal diseases	Rate	2005	174.86	336.07	62.83
145	All Population	DALYs (Disability-Adjusted Life Years)	Viet Nam	Female	60+ years	Periodontal diseases	Rate	1997	89.61	190.07	33.63
146	All Population	DALYs (Disability-Adjusted Life Years)	Lao People's Democratic Republic	Female	60+ years	Periodontal diseases	Rate	2007	84.86	184.74	30.18
147	All Population	DALYs (Disability-Adjusted Life Years)	Singapore	Female	60+ years	Periodontal diseases	Rate	2004	138.38	276.63	47.26
148	All Population	DALYs (Disability-Adjusted Life Years)	China	Female	60+ years	Periodontal diseases	Rate	2002	175.01	363.19	66.85
149	All Population	DALYs (Disability-Adjusted Life Years)	Cambodia	Female	60+ years	Periodontal diseases	Rate	2003	131.11	281.09	50.34
150	All Population	DALYs (Disability-Adjusted Life Years)	Brunei Darussalam	Female	60+ years	Periodontal diseases	Rate	2000	147.83	298.91	51.22
151	All Population	DALYs (Disability-Adjusted Life Years)	Association of Southeast Asian Nations	Female	60+ years	Periodontal diseases	Rate	2000	140.79	293.38	53.87
152	All Population	DALYs (Disability-Adjusted Life Years)	Cambodia	Female	60+ years	Periodontal diseases	Rate	2009	140.69	297.41	52.29
153	All Population	DALYs (Disability-Adjusted Life Years)	Viet Nam	Female	60+ years	Periodontal diseases	Rate	2002	91.28	183.86	34.78
154	All Population	DALYs (Disability-Adjusted Life Years)	Malaysia	Female	60+ years	Periodontal diseases	Rate	1996	144.17	302.55	53.42
155	All Population	DALYs (Disability-Adjusted Life Years)	Lao People's Democratic Republic	Female	60+ years	Periodontal diseases	Rate	2003	76.98	164.67	27.35
156	All Population	DALYs (Disability-Adjusted Life Years)	Myanmar	Female	60+ years	Periodontal diseases	Rate	1996	143.00	307.79	53.31
157	All Population	DALYs (Disability-Adjusted Life Years)	Association of Southeast Asian Nations	Female	60+ years	Periodontal diseases	Rate	2001	141.54	289.62	54.53
158	All Population	DALYs (Disability-Adjusted Life Years)	Malaysia	Female	60+ years	Periodontal diseases	Rate	1998	119.36	244.86	44.05
159	All Population	DALYs (Disability-Adjusted Life Years)	Singapore	Female	60+ years	Periodontal diseases	Rate	2003	137.80	273.16	47.30
160	All Population	DALYs (Disability-Adjusted Life Years)	Viet Nam	Female	60+ years	Periodontal diseases	Rate	1999	73.03	148.21	28.76
161	All Population	DALYs (Disability-Adjusted Life Years)	Viet Nam	Female	60+ years	Periodontal	Rate	2004	124.65	254.67	45.10

						diseases					
162	All Population	DALYs (Disability-Adjusted Life Years)	Brunei Darussalam	Female	60+ years	Periodontal diseases	Rate	2004	147.06	294.40	51.38
163	All Population	DALYs (Disability-Adjusted Life Years)	Association of Southeast Asian Nations	Female	60+ years	Periodontal diseases	Rate	1996	149.70	312.82	56.48
164	All Population	DALYs (Disability-Adjusted Life Years)	Myanmar	Female	60+ years	Periodontal diseases	Rate	1998	144.87	310.18	55.31
165	All Population	DALYs (Disability-Adjusted Life Years)	Malaysia	Female	60+ years	Periodontal diseases	Rate	2000	98.55	200.22	37.76
166	All Population	DALYs (Disability-Adjusted Life Years)	Indonesia	Female	60+ years	Periodontal diseases	Rate	2004	176.48	362.38	68.02
167	All Population	DALYs (Disability-Adjusted Life Years)	Viet Nam	Female	60+ years	Periodontal diseases	Rate	2005	131.74	272.01	47.40
168	All Population	DALYs (Disability-Adjusted Life Years)	Lao People's Democratic Republic	Female	60+ years	Periodontal diseases	Rate	2009	84.90	183.98	30.75
169	All Population	DALYs (Disability-Adjusted Life Years)	Association of Southeast Asian Nations	Female	60+ years	Periodontal diseases	Rate	1997	147.33	308.67	55.68
170	All Population	DALYs (Disability-Adjusted Life Years)	Viet Nam	Female	60+ years	Periodontal diseases	Rate	2003	109.28	224.90	40.59
171	All Population	DALYs (Disability-Adjusted Life Years)	Viet Nam	Female	60+ years	Periodontal diseases	Rate	1998	80.56	165.73	31.12
172	All Population	DALYs (Disability-Adjusted Life Years)	Singapore	Female	60+ years	Periodontal diseases	Rate	1997	142.70	286.24	51.57
173	All Population	DALYs (Disability-Adjusted Life Years)	Indonesia	Female	60+ years	Periodontal diseases	Rate	2005	176.53	364.22	67.98
174	All Population	DALYs (Disability-Adjusted Life Years)	Myanmar	Female	60+ years	Periodontal diseases	Rate	2003	139.38	284.16	49.27
175	All Population	DALYs (Disability-Adjusted Life Years)	China	Female	60+ years	Periodontal diseases	Rate	2004	176.01	356.87	66.88
176	All Population	DALYs (Disability-Adjusted Life Years)	Brunei Darussalam	Female	60+ years	Periodontal diseases	Rate	2003	147.24	295.73	51.19
177	All Population	DALYs (Disability-Adjusted Life Years)	Singapore	Female	60+ years	Periodontal diseases	Rate	2005	138.86	279.66	47.07
178	All Population	DALYs (Disability-Adjusted Life Years)	Malaysia	Female	60+ years	Periodontal diseases	Rate	2002	89.36	178.97	34.10
179	All Population	DALYs (Disability-Adjusted Life Years)	Cambodia	Female	60+ years	Periodontal diseases	Rate	2010	142.01	304.79	52.29
180	All Population	DALYs (Disability-Adjusted Life Years)	Cambodia	Female	60+ years	Periodontal diseases	Rate	2011	142.97	304.50	52.86
181	All Population	DALYs (Disability-Adjusted Life Years)	China	Female	60+ years	Periodontal diseases	Rate	2005	175.51	353.30	66.65

182	All Population	DALYs (Disability-Adjusted Life Years)	China	Female	60+ years	Periodontal diseases	Rate	1998	169.36	357.82	64.40
183	All Population	DALYs (Disability-Adjusted Life Years)	Association of Southeast Asian Nations	Female	60+ years	Periodontal diseases	Rate	2003	146.24	294.23	55.77
184	All Population	DALYs (Disability-Adjusted Life Years)	Indonesia	Female	60+ years	Periodontal diseases	Rate	2003	176.64	362.91	67.72
185	All Population	DALYs (Disability-Adjusted Life Years)	Singapore	Female	60+ years	Periodontal diseases	Rate	2000	136.47	269.56	46.46
186	All Population	DALYs (Disability-Adjusted Life Years)	Lao People's Democratic Republic	Female	60+ years	Periodontal diseases	Rate	2010	85.07	188.45	30.83
187	All Population	DALYs (Disability-Adjusted Life Years)	Lao People's Democratic Republic	Female	60+ years	Periodontal diseases	Rate	2011	85.86	186.92	30.95
188	All Population	DALYs (Disability-Adjusted Life Years)	Association of Southeast Asian Nations	Female	60+ years	Periodontal diseases	Rate	2004	148.45	295.62	55.95
189	All Population	DALYs (Disability-Adjusted Life Years)	Singapore	Female	60+ years	Periodontal diseases	Rate	2006	139.26	275.77	47.69
190	All Population	DALYs (Disability-Adjusted Life Years)	Philippines	Female	60+ years	Periodontal diseases	Rate	2006	96.12	193.84	36.02
191	All Population	DALYs (Disability-Adjusted Life Years)	Singapore	Female	60+ years	Periodontal diseases	Rate	2007	140.09	273.83	47.68
192	All Population	DALYs (Disability-Adjusted Life Years)	Thailand	Female	60+ years	Periodontal diseases	Rate	2006	176.35	338.83	63.21
193	All Population	DALYs (Disability-Adjusted Life Years)	Malaysia	Female	60+ years	Periodontal diseases	Rate	2004	83.03	159.87	30.89
194	All Population	DALYs (Disability-Adjusted Life Years)	Brunei Darussalam	Female	60+ years	Periodontal diseases	Rate	2006	146.98	293.09	51.25
195	All Population	DALYs (Disability-Adjusted Life Years)	Myanmar	Female	60+ years	Periodontal diseases	Rate	2007	133.80	267.77	48.66
196	All Population	DALYs (Disability-Adjusted Life Years)	Brunei Darussalam	Female	60+ years	Periodontal diseases	Rate	2005	146.94	295.34	51.61
197	All Population	DALYs (Disability-Adjusted Life Years)	Brunei Darussalam	Female	60+ years	Periodontal diseases	Rate	2007	147.12	293.77	50.56
198	All Population	DALYs (Disability-Adjusted Life Years)	China	Female	60+ years	Periodontal diseases	Rate	2003	175.78	360.60	66.93
199	All Population	DALYs (Disability-Adjusted Life Years)	Cambodia	Female	60+ years	Periodontal diseases	Rate	2012	144.03	307.92	53.47
200	All Population	DALYs (Disability-Adjusted Life Years)	Viet Nam	Female	60+ years	Periodontal diseases	Rate	2000	69.97	142.42	27.44
201	All Population	DALYs (Disability-Adjusted Life Years)	Indonesia	Female	60+ years	Periodontal diseases	Rate	1999	177.49	363.27	67.82

202	All Population	DALYs (Disability-Adjusted Life Years)	Myanmar	Female	60+ years	Periodontal diseases	Rate	2008	132.47	262.03	48.02
203	All Population	DALYs (Disability-Adjusted Life Years)	Myanmar	Female	60+ years	Periodontal diseases	Rate	2004	136.70	275.29	48.81
204	All Population	DALYs (Disability-Adjusted Life Years)	Lao People's Democratic Republic	Female	60+ years	Periodontal diseases	Rate	2012	87.46	192.59	31.55
205	All Population	DALYs (Disability-Adjusted Life Years)	Myanmar	Female	60+ years	Periodontal diseases	Rate	2005	135.53	276.99	49.02
206	All Population	DALYs (Disability-Adjusted Life Years)	Myanmar	Female	60+ years	Periodontal diseases	Rate	2006	134.84	274.45	48.95
207	All Population	DALYs (Disability-Adjusted Life Years)	Singapore	Female	60+ years	Periodontal diseases	Rate	2009	141.41	276.38	48.50
208	All Population	DALYs (Disability-Adjusted Life Years)	Indonesia	Female	60+ years	Periodontal diseases	Rate	2006	176.76	364.59	68.07
209	All Population	DALYs (Disability-Adjusted Life Years)	Cambodia	Female	60+ years	Periodontal diseases	Rate	2013	144.76	307.66	54.24
210	All Population	DALYs (Disability-Adjusted Life Years)	Lao People's Democratic Republic	Female	60+ years	Periodontal diseases	Rate	2013	89.28	198.57	31.62
211	All Population	DALYs (Disability-Adjusted Life Years)	Malaysia	Female	60+ years	Periodontal diseases	Rate	2007	101.69	196.85	36.19
212	All Population	DALYs (Disability-Adjusted Life Years)	Malaysia	Female	60+ years	Periodontal diseases	Rate	2005	82.22	159.09	29.42
213	All Population	DALYs (Disability-Adjusted Life Years)	China	Female	60+ years	Periodontal diseases	Rate	2007	165.08	334.02	62.46
214	All Population	DALYs (Disability-Adjusted Life Years)	Association of Southeast Asian Nations	Female	60+ years	Periodontal diseases	Rate	2007	150.41	298.04	56.05
215	All Population	DALYs (Disability-Adjusted Life Years)	Cambodia	Female	60+ years	Periodontal diseases	Rate	2014	145.65	311.12	54.60
216	All Population	DALYs (Disability-Adjusted Life Years)	Indonesia	Female	60+ years	Periodontal diseases	Rate	2001	177.09	364.09	67.58
217	All Population	DALYs (Disability-Adjusted Life Years)	Singapore	Female	60+ years	Periodontal diseases	Rate	1998	139.68	280.70	50.46
218	All Population	DALYs (Disability-Adjusted Life Years)	Indonesia	Female	60+ years	Periodontal diseases	Rate	2009	178.02	367.94	68.47
219	All Population	DALYs (Disability-Adjusted Life Years)	Singapore	Female	60+ years	Periodontal diseases	Rate	2008	140.75	276.74	48.04
220	All Population	DALYs (Disability-Adjusted Life Years)	Viet Nam	Female	60+ years	Periodontal diseases	Rate	2006	132.96	274.68	47.86
221	All Population	DALYs (Disability-Adjusted Life Years)	Viet Nam	Female	60+ years	Periodontal diseases	Rate	2010	135.39	272.53	49.20
222	All Population	DALYs (Disability-Adjusted Life Years)	Lao People's	Female	60+ years	Periodontal	Rate	2014	91.25	200.51	32.24

			Democratic Republic			diseases					
223	All Population	DALYs (Disability-Adjusted Life Years)	Philippines	Female	60+ years	Periodontal diseases	Rate	2007	91.87	183.56	34.03
224	All Population	DALYs (Disability-Adjusted Life Years)	China	Female	60+ years	Periodontal diseases	Rate	2008	157.01	318.98	59.32
225	All Population	DALYs (Disability-Adjusted Life Years)	China	Female	60+ years	Periodontal diseases	Rate	2006	172.05	346.22	65.12
226	All Population	DALYs (Disability-Adjusted Life Years)	Myanmar	Female	60+ years	Periodontal diseases	Rate	2010	131.30	254.72	46.70
227	All Population	DALYs (Disability-Adjusted Life Years)	Brunei Darussalam	Female	60+ years	Periodontal diseases	Rate	2009	147.40	292.85	49.96
228	All Population	DALYs (Disability-Adjusted Life Years)	Thailand	Female	60+ years	Periodontal diseases	Rate	2007	177.57	347.12	64.99
229	All Population	DALYs (Disability-Adjusted Life Years)	Indonesia	Female	60+ years	Periodontal diseases	Rate	2007	177.06	365.31	68.26
230	All Population	DALYs (Disability-Adjusted Life Years)	Indonesia	Female	60+ years	Periodontal diseases	Rate	2010	178.68	370.16	68.66
231	All Population	DALYs (Disability-Adjusted Life Years)	Indonesia	Female	60+ years	Periodontal diseases	Rate	2008	177.45	366.55	68.35
232	All Population	DALYs (Disability-Adjusted Life Years)	Association of Southeast Asian Nations	Female	60+ years	Periodontal diseases	Rate	2002	143.64	290.74	55.28
233	All Population	DALYs (Disability-Adjusted Life Years)	Association of Southeast Asian Nations	Female	60+ years	Periodontal diseases	Rate	2006	150.00	297.79	55.88
234	All Population	DALYs (Disability-Adjusted Life Years)	Myanmar	Female	60+ years	Periodontal diseases	Rate	2009	131.47	256.41	47.59
235	All Population	DALYs (Disability-Adjusted Life Years)	Brunei Darussalam	Female	60+ years	Periodontal diseases	Rate	2008	147.19	294.54	50.30
236	All Population	DALYs (Disability-Adjusted Life Years)	Singapore	Female	60+ years	Periodontal diseases	Rate	2010	141.91	272.08	48.23
237	All Population	DALYs (Disability-Adjusted Life Years)	Myanmar	Female	60+ years	Periodontal diseases	Rate	1999	145.75	311.69	55.59
238	All Population	DALYs (Disability-Adjusted Life Years)	Association of Southeast Asian Nations	Female	60+ years	Periodontal diseases	Rate	2008	150.75	298.93	56.10
239	All Population	DALYs (Disability-Adjusted Life Years)	China	Female	60+ years	Periodontal diseases	Rate	2009	150.56	307.65	56.88
240	All Population	DALYs (Disability-Adjusted Life Years)	Cambodia	Female	60+ years	Periodontal diseases	Rate	2015	146.37	319.59	54.87
241	All Population	DALYs (Disability-Adjusted Life Years)	Association of Southeast Asian Nations	Female	60+ years	Periodontal diseases	Rate	2009	151.05	300.08	56.48

242	All Population	DALYs (Disability-Adjusted Life Years)	Singapore	Female	60+ years	Periodontal diseases	Rate	2011	142.57	277.94	49.19
243	All Population	DALYs (Disability-Adjusted Life Years)	Singapore	Female	60+ years	Periodontal diseases	Rate	2012	143.33	278.97	49.18
244	All Population	DALYs (Disability-Adjusted Life Years)	Lao People's Democratic Republic	Female	60+ years	Periodontal diseases	Rate	2015	92.90	205.84	32.11
245	All Population	DALYs (Disability-Adjusted Life Years)	Malaysia	Female	60+ years	Periodontal diseases	Rate	2006	88.38	172.27	31.45
246	All Population	DALYs (Disability-Adjusted Life Years)	Malaysia	Female	60+ years	Periodontal diseases	Rate	2003	85.58	170.36	32.28
247	All Population	DALYs (Disability-Adjusted Life Years)	China	Female	60+ years	Periodontal diseases	Rate	2010	147.76	303.53	55.74
248	All Population	DALYs (Disability-Adjusted Life Years)	Association of Southeast Asian Nations	Female	60+ years	Periodontal diseases	Rate	2005	149.59	295.91	55.75
249	All Population	DALYs (Disability-Adjusted Life Years)	Brunei Darussalam	Female	60+ years	Periodontal diseases	Rate	2010	147.41	291.72	49.95
250	All Population	DALYs (Disability-Adjusted Life Years)	Viet Nam	Female	60+ years	Periodontal diseases	Rate	2012	134.37	270.68	46.97
251	All Population	DALYs (Disability-Adjusted Life Years)	Philippines	Female	60+ years	Periodontal diseases	Rate	2008	86.90	172.09	31.80
252	All Population	DALYs (Disability-Adjusted Life Years)	Viet Nam	Female	60+ years	Periodontal diseases	Rate	2011	135.14	270.96	48.29
253	All Population	DALYs (Disability-Adjusted Life Years)	Myanmar	Female	60+ years	Periodontal diseases	Rate	2011	131.64	256.01	46.76
254	All Population	DALYs (Disability-Adjusted Life Years)	Viet Nam	Female	60+ years	Periodontal diseases	Rate	2007	133.97	275.82	48.85
255	All Population	DALYs (Disability-Adjusted Life Years)	Malaysia	Female	60+ years	Periodontal diseases	Rate	2009	131.45	255.99	46.74
256	All Population	DALYs (Disability-Adjusted Life Years)	Brunei Darussalam	Female	60+ years	Periodontal diseases	Rate	2011	147.44	291.82	50.08
257	All Population	DALYs (Disability-Adjusted Life Years)	China	Female	60+ years	Periodontal diseases	Rate	2011	152.32	309.10	57.39
258	All Population	DALYs (Disability-Adjusted Life Years)	Thailand	Female	60+ years	Periodontal diseases	Rate	2008	178.63	350.63	65.38
259	All Population	DALYs (Disability-Adjusted Life Years)	Malaysia	Female	60+ years	Periodontal diseases	Rate	2001	93.74	189.35	36.51
260	All Population	DALYs (Disability-Adjusted Life Years)	Indonesia	Female	60+ years	Periodontal diseases	Rate	2012	180.26	372.06	69.30
261	All Population	DALYs (Disability-Adjusted Life Years)	Viet Nam	Female	60+ years	Periodontal diseases	Rate	2013	133.53	267.23	46.03
262	All Population	DALYs (Disability-Adjusted Life Years)	Indonesia	Female	60+ years	Periodontal diseases	Rate	2013	181.20	372.01	69.57

263	All Population	DALYs (Disability-Adjusted Life Years)	Association of Southeast Asian Nations	Female	60+ years	Periodontal diseases	Rate	2010	151.37	301.08	56.69
264	All Population	DALYs (Disability-Adjusted Life Years)	Singapore	Female	60+ years	Periodontal diseases	Rate	2013	144.11	276.54	49.63
265	All Population	DALYs (Disability-Adjusted Life Years)	Cambodia	Female	60+ years	Periodontal diseases	Rate	2016	147.25	321.23	55.68
266	All Population	DALYs (Disability-Adjusted Life Years)	Malaysia	Female	60+ years	Periodontal diseases	Rate	2008	117.87	229.03	41.69
267	All Population	DALYs (Disability-Adjusted Life Years)	Association of Southeast Asian Nations	Female	60+ years	Periodontal diseases	Rate	2011	151.42	299.34	56.76
268	All Population	DALYs (Disability-Adjusted Life Years)	China	Female	60+ years	Periodontal diseases	Rate	2012	163.23	328.05	61.30
269	All Population	DALYs (Disability-Adjusted Life Years)	Lao People's Democratic Republic	Female	60+ years	Periodontal diseases	Rate	2016	94.17	210.30	33.04
270	All Population	DALYs (Disability-Adjusted Life Years)	Malaysia	Female	60+ years	Periodontal diseases	Rate	2010	137.35	270.13	49.04
271	All Population	DALYs (Disability-Adjusted Life Years)	Viet Nam	Female	60+ years	Periodontal diseases	Rate	2009	135.27	271.49	49.18
272	All Population	DALYs (Disability-Adjusted Life Years)	Singapore	Female	60+ years	Periodontal diseases	Rate	2014	144.63	278.88	49.66
273	All Population	DALYs (Disability-Adjusted Life Years)	Myanmar	Female	60+ years	Periodontal diseases	Rate	2012	132.80	257.95	47.34
274	All Population	DALYs (Disability-Adjusted Life Years)	Brunei Darussalam	Female	60+ years	Periodontal diseases	Rate	2012	147.60	294.66	50.49
275	All Population	DALYs (Disability-Adjusted Life Years)	Indonesia	Female	60+ years	Periodontal diseases	Rate	2014	182.20	371.33	69.93
276	All Population	DALYs (Disability-Adjusted Life Years)	Viet Nam	Female	60+ years	Periodontal diseases	Rate	2014	132.87	264.84	45.04
277	All Population	DALYs (Disability-Adjusted Life Years)	Malaysia	Female	60+ years	Periodontal diseases	Rate	2012	136.04	261.32	48.56
278	All Population	DALYs (Disability-Adjusted Life Years)	Viet Nam	Female	60+ years	Periodontal diseases	Rate	2001	76.29	154.00	29.95
279	All Population	DALYs (Disability-Adjusted Life Years)	Indonesia	Female	60+ years	Periodontal diseases	Rate	2011	179.33	371.08	68.95
280	All Population	DALYs (Disability-Adjusted Life Years)	Malaysia	Female	60+ years	Periodontal diseases	Rate	2013	134.94	258.10	47.39
281	All Population	DALYs (Disability-Adjusted Life Years)	Viet Nam	Female	60+ years	Periodontal diseases	Rate	2008	134.81	273.99	48.92
282	All Population	DALYs (Disability-Adjusted Life Years)	Singapore	Female	60+ years	Periodontal diseases	Rate	2015	145.12	285.77	50.10
283	All Population	DALYs (Disability-Adjusted Life Years)	Cambodia	Female	60+ years	Periodontal	Rate	2017	148.36	320.07	56.27

						diseases					
284	All Population	DALYs (Disability-Adjusted Life Years)	Malaysia	Female	60+ years	Periodontal diseases	Rate	2014	134.06	253.91	46.43
285	All Population	DALYs (Disability-Adjusted Life Years)	Malaysia	Female	60+ years	Periodontal diseases	Rate	2015	133.69	253.61	46.11
286	All Population	DALYs (Disability-Adjusted Life Years)	Lao People's Democratic Republic	Female	60+ years	Periodontal diseases	Rate	2017	95.42	215.93	33.96
287	All Population	DALYs (Disability-Adjusted Life Years)	Association of Southeast Asian Nations	Female	60+ years	Periodontal diseases	Rate	2012	151.31	298.37	56.85
288	All Population	DALYs (Disability-Adjusted Life Years)	Indonesia	Female	60+ years	Periodontal diseases	Rate	2015	182.97	372.55	70.27
289	All Population	DALYs (Disability-Adjusted Life Years)	Malaysia	Female	60+ years	Periodontal diseases	Rate	2011	136.92	264.61	49.00
290	All Population	DALYs (Disability-Adjusted Life Years)	China	Female	60+ years	Periodontal diseases	Rate	2013	176.33	350.34	66.31
291	All Population	DALYs (Disability-Adjusted Life Years)	Viet Nam	Female	60+ years	Periodontal diseases	Rate	2015	132.71	261.99	44.06
292	All Population	DALYs (Disability-Adjusted Life Years)	Brunei Darussalam	Female	60+ years	Periodontal diseases	Rate	2013	147.59	292.59	50.32
293	All Population	DALYs (Disability-Adjusted Life Years)	Indonesia	Female	60+ years	Periodontal diseases	Rate	2016	183.55	375.89	70.69
294	All Population	DALYs (Disability-Adjusted Life Years)	Singapore	Female	60+ years	Periodontal diseases	Rate	2016	145.44	286.34	50.52
295	All Population	DALYs (Disability-Adjusted Life Years)	Philippines	Female	60+ years	Periodontal diseases	Rate	2009	82.77	162.62	29.95
296	All Population	DALYs (Disability-Adjusted Life Years)	Thailand	Female	60+ years	Periodontal diseases	Rate	2009	179.36	351.27	65.85
297	All Population	DALYs (Disability-Adjusted Life Years)	Malaysia	Female	60+ years	Periodontal diseases	Rate	2016	134.09	254.10	45.91
298	All Population	DALYs (Disability-Adjusted Life Years)	Cambodia	Female	60+ years	Periodontal diseases	Rate	2018	149.43	325.78	56.68
299	All Population	DALYs (Disability-Adjusted Life Years)	Myanmar	Female	60+ years	Periodontal diseases	Rate	2013	134.32	265.76	47.55
300	All Population	DALYs (Disability-Adjusted Life Years)	Lao People's Democratic Republic	Female	60+ years	Periodontal diseases	Rate	2018	96.54	216.83	34.65
301	All Population	DALYs (Disability-Adjusted Life Years)	Myanmar	Female	60+ years	Periodontal diseases	Rate	2014	135.73	269.04	48.46
302	All Population	DALYs (Disability-Adjusted Life Years)	Association of Southeast Asian Nations	Female	60+ years	Periodontal diseases	Rate	2013	151.14	296.41	56.73
303	All Population	DALYs (Disability-Adjusted Life Years)	Viet Nam	Female	60+ years	Periodontal	Rate	2016	133.60	262.06	44.81

						diseases					
304	All Population	DALYs (Disability-Adjusted Life Years)	China	Female	60+ years	Periodontal diseases	Rate	2014	187.28	368.80	70.57
305	All Population	DALYs (Disability-Adjusted Life Years)	Indonesia	Female	60+ years	Periodontal diseases	Rate	2017	184.13	378.59	70.78
306	All Population	DALYs (Disability-Adjusted Life Years)	Singapore	Female	60+ years	Periodontal diseases	Rate	2017	145.76	292.62	50.51
307	All Population	DALYs (Disability-Adjusted Life Years)	Philippines	Female	60+ years	Periodontal diseases	Rate	2010	80.92	157.63	29.04
308	All Population	DALYs (Disability-Adjusted Life Years)	Brunei Darussalam	Female	60+ years	Periodontal diseases	Rate	2014	147.67	293.97	50.23
309	All Population	DALYs (Disability-Adjusted Life Years)	Malaysia	Female	60+ years	Periodontal diseases	Rate	2017	135.09	251.08	46.88
310	All Population	DALYs (Disability-Adjusted Life Years)	Thailand	Female	60+ years	Periodontal diseases	Rate	2010	180.02	351.03	66.43
311	All Population	DALYs (Disability-Adjusted Life Years)	Viet Nam	Female	60+ years	Periodontal diseases	Rate	2017	135.43	263.44	45.77
312	All Population	DALYs (Disability-Adjusted Life Years)	Viet Nam	Female	60+ years	Periodontal diseases	Rate	2018	137.58	265.41	46.85
313	All Population	DALYs (Disability-Adjusted Life Years)	Indonesia	Female	60+ years	Periodontal diseases	Rate	2018	184.55	379.01	70.74
314	All Population	DALYs (Disability-Adjusted Life Years)	Association of Southeast Asian Nations	Female	60+ years	Periodontal diseases	Rate	2014	151.13	296.61	56.79
315	All Population	DALYs (Disability-Adjusted Life Years)	Myanmar	Female	60+ years	Periodontal diseases	Rate	2015	137.19	270.15	48.30
316	All Population	DALYs (Disability-Adjusted Life Years)	China	Female	60+ years	Periodontal diseases	Rate	2015	191.96	375.57	72.67
317	All Population	DALYs (Disability-Adjusted Life Years)	Malaysia	Female	60+ years	Periodontal diseases	Rate	2018	136.32	253.58	47.94
318	All Population	DALYs (Disability-Adjusted Life Years)	Singapore	Female	60+ years	Periodontal diseases	Rate	2018	145.97	295.19	50.14
319	All Population	DALYs (Disability-Adjusted Life Years)	Brunei Darussalam	Female	60+ years	Periodontal diseases	Rate	2015	147.52	297.98	49.77
320	All Population	DALYs (Disability-Adjusted Life Years)	Cambodia	Female	60+ years	Periodontal diseases	Rate	2019	150.14	329.94	57.35
321	All Population	DALYs (Disability-Adjusted Life Years)	Lao People's Democratic Republic	Female	60+ years	Periodontal diseases	Rate	2019	97.71	218.81	35.18
322	All Population	DALYs (Disability-Adjusted Life Years)	Association of Southeast Asian Nations	Female	60+ years	Periodontal diseases	Rate	2015	151.41	296.86	57.01
323	All Population	DALYs (Disability-Adjusted Life Years)	China	Female	60+ years	Periodontal diseases	Rate	2016	188.94	369.12	71.42

324	All Population	DALYs (Disability-Adjusted Life Years)	Myanmar	Female	60+ years	Periodontal diseases	Rate	2016	138.82	277.29	48.31
325	All Population	DALYs (Disability-Adjusted Life Years)	Brunei Darussalam	Female	60+ years	Periodontal diseases	Rate	2016	146.88	296.10	49.43
326	All Population	DALYs (Disability-Adjusted Life Years)	Philippines	Female	60+ years	Periodontal diseases	Rate	2011	79.44	154.59	28.03
327	All Population	DALYs (Disability-Adjusted Life Years)	Thailand	Female	60+ years	Periodontal diseases	Rate	2011	180.52	352.42	66.60
328	All Population	DALYs (Disability-Adjusted Life Years)	Viet Nam	Female	60+ years	Periodontal diseases	Rate	2019	139.05	271.55	47.69
329	All Population	DALYs (Disability-Adjusted Life Years)	Indonesia	Female	60+ years	Periodontal diseases	Rate	2019	184.99	381.92	70.64
330	All Population	DALYs (Disability-Adjusted Life Years)	Singapore	Female	60+ years	Periodontal diseases	Rate	2019	146.15	295.64	50.40
331	All Population	DALYs (Disability-Adjusted Life Years)	Malaysia	Female	60+ years	Periodontal diseases	Rate	2019	136.69	255.56	47.74
332	All Population	DALYs (Disability-Adjusted Life Years)	Cambodia	Female	60+ years	Periodontal diseases	Rate	2020	151.02	329.35	57.16
333	All Population	DALYs (Disability-Adjusted Life Years)	Lao People's Democratic Republic	Female	60+ years	Periodontal diseases	Rate	2020	98.84	222.02	35.41
334	All Population	DALYs (Disability-Adjusted Life Years)	Association of Southeast Asian Nations	Female	60+ years	Periodontal diseases	Rate	2016	152.13	298.83	57.17
335	All Population	DALYs (Disability-Adjusted Life Years)	Myanmar	Female	60+ years	Periodontal diseases	Rate	2017	140.78	281.91	48.87
336	All Population	DALYs (Disability-Adjusted Life Years)	China	Female	60+ years	Periodontal diseases	Rate	2017	182.14	356.05	68.76
337	All Population	DALYs (Disability-Adjusted Life Years)	Brunei Darussalam	Female	60+ years	Periodontal diseases	Rate	2017	145.91	292.72	50.14
338	All Population	DALYs (Disability-Adjusted Life Years)	Viet Nam	Female	60+ years	Periodontal diseases	Rate	2020	139.93	270.55	47.98
339	All Population	DALYs (Disability-Adjusted Life Years)	Indonesia	Female	60+ years	Periodontal diseases	Rate	2020	185.36	381.01	71.34
340	All Population	DALYs (Disability-Adjusted Life Years)	Singapore	Female	60+ years	Periodontal diseases	Rate	2020	146.61	294.12	49.84
341	All Population	DALYs (Disability-Adjusted Life Years)	Malaysia	Female	60+ years	Periodontal diseases	Rate	2020	136.31	259.29	47.99
342	All Population	DALYs (Disability-Adjusted Life Years)	Cambodia	Female	60+ years	Periodontal diseases	Rate	2021	152.25	331.48	56.65
343	All Population	DALYs (Disability-Adjusted Life Years)	Thailand	Female	60+ years	Periodontal diseases	Rate	2012	180.95	357.13	67.55
344	All Population	DALYs (Disability-Adjusted Life Years)	Philippines	Female	60+ years	Periodontal diseases	Rate	2012	76.20	147.88	26.63

345	All Population	DALYs (Disability-Adjusted Life Years)	Lao People's Democratic Republic	Female	60+ years	Periodontal diseases	Rate	2021	100.00	223.80	36.36
346	All Population	DALYs (Disability-Adjusted Life Years)	Myanmar	Female	60+ years	Periodontal diseases	Rate	2018	142.32	284.58	49.51
347	All Population	DALYs (Disability-Adjusted Life Years)	China	Female	60+ years	Periodontal diseases	Rate	2018	174.88	342.31	66.00
348	All Population	DALYs (Disability-Adjusted Life Years)	Association of Southeast Asian Nations	Female	60+ years	Periodontal diseases	Rate	2017	153.13	300.28	57.47
349	All Population	DALYs (Disability-Adjusted Life Years)	Indonesia	Female	60+ years	Periodontal diseases	Rate	2021	186.05	383.19	71.07
350	All Population	DALYs (Disability-Adjusted Life Years)	Viet Nam	Female	60+ years	Periodontal diseases	Rate	2021	140.36	270.98	47.97
351	All Population	DALYs (Disability-Adjusted Life Years)	Singapore	Female	60+ years	Periodontal diseases	Rate	2021	147.14	291.80	51.91
352	All Population	DALYs (Disability-Adjusted Life Years)	Malaysia	Female	60+ years	Periodontal diseases	Rate	2021	136.70	255.94	47.29
353	All Population	DALYs (Disability-Adjusted Life Years)	Brunei Darussalam	Female	60+ years	Periodontal diseases	Rate	2018	145.03	291.17	49.99
354	All Population	DALYs (Disability-Adjusted Life Years)	Cambodia	Female	60+ years	Periodontal diseases	Rate	2022	151.84	330.80	56.59
355	All Population	DALYs (Disability-Adjusted Life Years)	Lao People's Democratic Republic	Female	60+ years	Periodontal diseases	Rate	2022	99.95	223.83	36.26
356	All Population	DALYs (Disability-Adjusted Life Years)	Indonesia	Female	60+ years	Periodontal diseases	Rate	2022	185.75	381.21	70.49
357	All Population	DALYs (Disability-Adjusted Life Years)	Viet Nam	Female	60+ years	Periodontal diseases	Rate	2022	141.34	279.55	48.56
358	All Population	DALYs (Disability-Adjusted Life Years)	Singapore	Female	60+ years	Periodontal diseases	Rate	2022	146.15	283.44	50.22
359	All Population	DALYs (Disability-Adjusted Life Years)	Philippines	Female	60+ years	Periodontal diseases	Rate	2013	72.38	139.76	25.01
360	All Population	DALYs (Disability-Adjusted Life Years)	Malaysia	Female	60+ years	Periodontal diseases	Rate	2022	136.47	257.51	46.86
361	All Population	DALYs (Disability-Adjusted Life Years)	Thailand	Female	60+ years	Periodontal diseases	Rate	2013	181.32	356.49	67.89
362	All Population	DALYs (Disability-Adjusted Life Years)	Indonesia	Female	60+ years	Periodontal diseases	Rate	2023	183.77	376.78	69.74
363	All Population	DALYs (Disability-Adjusted Life Years)	Malaysia	Female	60+ years	Periodontal diseases	Rate	2023	135.03	257.03	47.05
364	All Population	DALYs (Disability-Adjusted Life Years)	Cambodia	Female	60+ years	Periodontal diseases	Rate	2023	149.71	324.87	55.59
365	All Population	DALYs (Disability-Adjusted Life Years)	Lao People's	Female	60+ years	Periodontal	Rate	2023	98.59	223.93	35.61

			Democratic Republic			diseases					
366	All Population	DALYs (Disability-Adjusted Life Years)	Viet Nam	Female	60+ years	Periodontal diseases	Rate	2023	139.54	276.43	47.85
367	All Population	DALYs (Disability-Adjusted Life Years)	Singapore	Female	60+ years	Periodontal diseases	Rate	2023	145.35	280.84	50.71
368	All Population	DALYs (Disability-Adjusted Life Years)	Myanmar	Female	60+ years	Periodontal diseases	Rate	2019	143.26	289.51	49.94
369	All Population	DALYs (Disability-Adjusted Life Years)	China	Female	60+ years	Periodontal diseases	Rate	2019	170.53	335.01	64.45
370	All Population	DALYs (Disability-Adjusted Life Years)	Association of Southeast Asian Nations	Female	60+ years	Periodontal diseases	Rate	2018	154.12	301.69	57.88
371	All Population	DALYs (Disability-Adjusted Life Years)	Brunei Darussalam	Female	60+ years	Periodontal diseases	Rate	2019	144.55	289.68	49.64
372	All Population	DALYs (Disability-Adjusted Life Years)	Philippines	Female	60+ years	Periodontal diseases	Rate	2014	69.22	134.04	23.69
373	All Population	DALYs (Disability-Adjusted Life Years)	Thailand	Female	60+ years	Periodontal diseases	Rate	2014	181.79	357.87	67.82
374	All Population	DALYs (Disability-Adjusted Life Years)	Association of Southeast Asian Nations	Female	60+ years	Periodontal diseases	Rate	2019	154.83	303.31	58.10
375	All Population	DALYs (Disability-Adjusted Life Years)	China	Female	60+ years	Periodontal diseases	Rate	2020	168.86	330.13	63.39
376	All Population	DALYs (Disability-Adjusted Life Years)	Brunei Darussalam	Female	60+ years	Periodontal diseases	Rate	2020	145.18	289.44	49.48
377	All Population	DALYs (Disability-Adjusted Life Years)	Myanmar	Female	60+ years	Periodontal diseases	Rate	2020	142.65	280.09	49.02
378	All Population	DALYs (Disability-Adjusted Life Years)	Philippines	Female	60+ years	Periodontal diseases	Rate	2015	67.94	131.53	23.08
379	All Population	DALYs (Disability-Adjusted Life Years)	China	Female	60+ years	Periodontal diseases	Rate	2021	168.37	329.76	62.96
380	All Population	DALYs (Disability-Adjusted Life Years)	Association of Southeast Asian Nations	Female	60+ years	Periodontal diseases	Rate	2020	155.19	303.54	59.19
381	All Population	DALYs (Disability-Adjusted Life Years)	Myanmar	Female	60+ years	Periodontal diseases	Rate	2021	143.52	290.17	50.37
382	All Population	DALYs (Disability-Adjusted Life Years)	Thailand	Female	60+ years	Periodontal diseases	Rate	2015	182.20	362.25	68.76
383	All Population	DALYs (Disability-Adjusted Life Years)	Brunei Darussalam	Female	60+ years	Periodontal diseases	Rate	2021	144.17	293.63	49.25
384	All Population	DALYs (Disability-Adjusted Life Years)	Myanmar	Female	60+ years	Periodontal diseases	Rate	2022	145.25	288.05	51.38
385	All Population	DALYs (Disability-Adjusted Life Years)	Association of	Female	60+ years	Periodontal	Rate	2021	155.62	303.57	58.99

			Southeast Asian Nations			diseases					
386	All Population	DALYs (Disability-Adjusted Life Years)	China	Female	60+ years	Periodontal diseases	Rate	2022	167.34	328.44	62.35
387	All Population	DALYs (Disability-Adjusted Life Years)	Brunei Darussalam	Female	60+ years	Periodontal diseases	Rate	2022	143.29	286.75	48.53
388	All Population	DALYs (Disability-Adjusted Life Years)	Myanmar	Female	60+ years	Periodontal diseases	Rate	2023	144.00	285.80	50.33
389	All Population	DALYs (Disability-Adjusted Life Years)	Association of Southeast Asian Nations	Female	60+ years	Periodontal diseases	Rate	2022	156.22	306.67	58.93
390	All Population	DALYs (Disability-Adjusted Life Years)	China	Female	60+ years	Periodontal diseases	Rate	2023	164.78	323.63	61.39
391	All Population	DALYs (Disability-Adjusted Life Years)	Brunei Darussalam	Female	60+ years	Periodontal diseases	Rate	2023	142.45	286.23	48.62
392	All Population	DALYs (Disability-Adjusted Life Years)	Philippines	Female	60+ years	Periodontal diseases	Rate	2016	68.09	132.01	23.21
393	All Population	DALYs (Disability-Adjusted Life Years)	Philippines	Female	60+ years	Periodontal diseases	Rate	2017	68.38	132.59	23.36
394	All Population	DALYs (Disability-Adjusted Life Years)	Thailand	Female	60+ years	Periodontal diseases	Rate	2016	182.97	362.16	68.60
395	All Population	DALYs (Disability-Adjusted Life Years)	Thailand	Female	60+ years	Periodontal diseases	Rate	2017	183.82	354.72	68.33
396	All Population	DALYs (Disability-Adjusted Life Years)	Association of Southeast Asian Nations	Female	60+ years	Periodontal diseases	Rate	2023	154.60	303.33	58.35
397	All Population	DALYs (Disability-Adjusted Life Years)	Philippines	Female	60+ years	Periodontal diseases	Rate	2018	68.65	133.31	23.56
398	All Population	DALYs (Disability-Adjusted Life Years)	Thailand	Female	60+ years	Periodontal diseases	Rate	2018	184.69	355.11	68.46
399	All Population	DALYs (Disability-Adjusted Life Years)	Philippines	Female	60+ years	Periodontal diseases	Rate	2019	68.77	133.74	23.71
400	All Population	DALYs (Disability-Adjusted Life Years)	Thailand	Female	60+ years	Periodontal diseases	Rate	2019	185.19	356.39	68.29
401	All Population	DALYs (Disability-Adjusted Life Years)	Thailand	Female	60+ years	Periodontal diseases	Rate	2020	185.39	356.51	68.60
402	All Population	DALYs (Disability-Adjusted Life Years)	Philippines	Female	60+ years	Periodontal diseases	Rate	2020	68.56	134.19	23.43
403	All Population	DALYs (Disability-Adjusted Life Years)	Philippines	Female	60+ years	Periodontal diseases	Rate	2021	68.26	133.87	23.59
404	All Population	DALYs (Disability-Adjusted Life Years)	Thailand	Female	60+ years	Periodontal diseases	Rate	2021	184.87	357.37	68.14
405	All Population	DALYs (Disability-Adjusted Life Years)	Philippines	Female	60+ years	Periodontal diseases	Rate	2022	68.83	134.41	23.74

406	All Population	DALYs (Disability-Adjusted Life Years)	Thailand	Female	60+ years	Periodontal diseases	Rate	2022	185.80	357.95	70.09
407	All Population	DALYs (Disability-Adjusted Life Years)	Philippines	Female	60+ years	Periodontal diseases	Rate	2023	67.85	132.05	23.44
408	All Population	DALYs (Disability-Adjusted Life Years)	Thailand	Female	60+ years	Periodontal diseases	Rate	2023	183.93	355.41	69.23

Supplemental Table S5: Trend Analysis of Edentulism from 1990 to 2023

	population_group_name	measure_name	location_name	sex_name	age_name	cause_name	metric_name	year	val	upper	lower
1	All Population	DALYs (Disability-Adjusted Life Years)	Cambodia	Female	60+ years	Edentulism	Rate	1991	726.12	1039.75	460.65
2	All Population	DALYs (Disability-Adjusted Life Years)	Philippines	Female	60+ years	Edentulism	Rate	1990	945.63	1340.91	609.39
3	All Population	DALYs (Disability-Adjusted Life Years)	Thailand	Female	60+ years	Edentulism	Rate	1990	667.09	954.05	424.26
4	All Population	DALYs (Disability-Adjusted Life Years)	Myanmar	Female	60+ years	Edentulism	Rate	1990	486.46	698.65	303.97
5	All Population	DALYs (Disability-Adjusted Life Years)	Malaysia	Female	60+ years	Edentulism	Rate	1991	939.92	1336.82	618.44
6	All Population	DALYs (Disability-Adjusted Life Years)	China	Female	60+ years	Edentulism	Rate	1990	636.17	931.44	416.25
7	All Population	DALYs (Disability-Adjusted Life Years)	Brunei Darussalam	Female	60+ years	Edentulism	Rate	1990	290.09	412.94	189.21
8	All Population	DALYs (Disability-Adjusted Life Years)	Indonesia	Female	60+ years	Edentulism	Rate	1991	700.44	999.61	447.75
9	All Population	DALYs (Disability-Adjusted Life Years)	Singapore	Female	60+ years	Edentulism	Rate	1991	329.90	469.44	212.56
10	All Population	DALYs (Disability-Adjusted Life Years)	Viet Nam	Female	60+ years	Edentulism	Rate	1991	705.69	1006.86	455.30
11	All Population	DALYs (Disability-Adjusted Life Years)	China	Female	60+ years	Edentulism	Rate	1991	629.68	918.49	411.66
12	All Population	DALYs (Disability-Adjusted Life Years)	Cambodia	Female	60+ years	Edentulism	Rate	1990	726.95	1037.20	454.46
13	All Population	DALYs (Disability-Adjusted Life Years)	Lao People's Democratic Republic	Female	60+ years	Edentulism	Rate	1992	498.89	725.44	316.44
14	All Population	DALYs (Disability-Adjusted Life Years)	Singapore	Female	60+ years	Edentulism	Rate	1990	332.75	476.90	213.82
15	All Population	DALYs (Disability-Adjusted Life Years)	Association of Southeast Asian Nations	Female	60+ years	Edentulism	Rate	1991	706.30	1005.62	451.24
16	All Population	DALYs (Disability-Adjusted Life Years)	Myanmar	Female	60+ years	Edentulism	Rate	1991	486.02	699.26	303.24
17	All Population	DALYs (Disability-Adjusted Life Years)	Viet Nam	Female	60+ years	Edentulism	Rate	1990	705.80	1010.33	459.56
18	All Population	DALYs (Disability-Adjusted Life Years)	Lao People's Democratic Republic	Female	60+ years	Edentulism	Rate	1991	501.50	725.85	318.47
19	All Population	DALYs (Disability-Adjusted Life Years)	Philippines	Female	60+ years	Edentulism	Rate	1991	946.00	1342.46	608.34
20	All Population	DALYs (Disability-Adjusted Life	Thailand	Female	60+ years	Edentulism	Rate	1991	660.34	949.21	421.24

		Years)									
21	All Population	DALYs (Disability-Adjusted Life Years)	Indonesia	Female	60+ years	Edentulism	Rate	1993	696.42	999.08	443.35
22	All Population	DALYs (Disability-Adjusted Life Years)	Indonesia	Female	60+ years	Edentulism	Rate	1990	704.13	1002.41	450.94
23	All Population	DALYs (Disability-Adjusted Life Years)	Association of Southeast Asian Nations	Female	60+ years	Edentulism	Rate	1990	709.39	1013.26	453.33
24	All Population	DALYs (Disability-Adjusted Life Years)	Brunei Darussalam	Female	60+ years	Edentulism	Rate	1991	291.05	415.13	188.10
25	All Population	DALYs (Disability-Adjusted Life Years)	Myanmar	Female	60+ years	Edentulism	Rate	1992	486.19	692.88	303.32
26	All Population	DALYs (Disability-Adjusted Life Years)	China	Female	60+ years	Edentulism	Rate	1992	619.36	902.67	404.84
27	All Population	DALYs (Disability-Adjusted Life Years)	Singapore	Female	60+ years	Edentulism	Rate	1993	327.60	476.95	212.91
28	All Population	DALYs (Disability-Adjusted Life Years)	Singapore	Female	60+ years	Edentulism	Rate	1994	325.51	475.51	211.89
29	All Population	DALYs (Disability-Adjusted Life Years)	Singapore	Female	60+ years	Edentulism	Rate	1992	328.72	473.49	212.05
30	All Population	DALYs (Disability-Adjusted Life Years)	China	Female	60+ years	Edentulism	Rate	1993	605.19	877.87	393.30
31	All Population	DALYs (Disability-Adjusted Life Years)	Philippines	Female	60+ years	Edentulism	Rate	1992	946.14	1342.79	608.22
32	All Population	DALYs (Disability-Adjusted Life Years)	Brunei Darussalam	Female	60+ years	Edentulism	Rate	1993	286.58	414.78	185.05
33	All Population	DALYs (Disability-Adjusted Life Years)	Philippines	Female	60+ years	Edentulism	Rate	1993	945.16	1343.77	606.53
34	All Population	DALYs (Disability-Adjusted Life Years)	Association of Southeast Asian Nations	Female	60+ years	Edentulism	Rate	1992	703.62	1003.81	449.68
35	All Population	DALYs (Disability-Adjusted Life Years)	Myanmar	Female	60+ years	Edentulism	Rate	1993	486.31	691.38	303.58
36	All Population	DALYs (Disability-Adjusted Life Years)	Indonesia	Female	60+ years	Edentulism	Rate	1994	695.22	998.07	440.16
37	All Population	DALYs (Disability-Adjusted Life Years)	Cambodia	Female	60+ years	Edentulism	Rate	1994	721.51	1037.14	453.78
38	All Population	DALYs (Disability-Adjusted Life Years)	Cambodia	Female	60+ years	Edentulism	Rate	1992	724.01	1041.04	451.61
39	All Population	DALYs (Disability-Adjusted Life Years)	Cambodia	Female	60+ years	Edentulism	Rate	1993	723.14	1035.83	455.71
40	All Population	DALYs (Disability-Adjusted Life Years)	Cambodia	Female	60+ years	Edentulism	Rate	1995	722.35	1038.60	455.23
41	All Population	DALYs (Disability-Adjusted Life	Brunei	Female	60+ years	Edentulism	Rate	1992	290.46	413.19	190.12

		Years)	Darussalam								
42	All Population	DALYs (Disability-Adjusted Life Years)	Viet Nam	Female	60+ years	Edentulism	Rate	1994	695.01	1001.12	442.66
43	All Population	DALYs (Disability-Adjusted Life Years)	Lao People's Democratic Republic	Female	60+ years	Edentulism	Rate	1990	505.64	728.06	314.02
44	All Population	DALYs (Disability-Adjusted Life Years)	Indonesia	Female	60+ years	Edentulism	Rate	1992	698.17	998.41	444.97
45	All Population	DALYs (Disability-Adjusted Life Years)	Cambodia	Female	60+ years	Edentulism	Rate	1996	724.88	1038.90	462.01
46	All Population	DALYs (Disability-Adjusted Life Years)	Indonesia	Female	60+ years	Edentulism	Rate	1995	695.45	998.47	440.51
47	All Population	DALYs (Disability-Adjusted Life Years)	Cambodia	Female	60+ years	Edentulism	Rate	1997	727.15	1035.85	461.96
48	All Population	DALYs (Disability-Adjusted Life Years)	Malaysia	Female	60+ years	Edentulism	Rate	1993	925.29	1331.08	601.75
49	All Population	DALYs (Disability-Adjusted Life Years)	Lao People's Democratic Republic	Female	60+ years	Edentulism	Rate	1994	492.97	715.51	316.08
50	All Population	DALYs (Disability-Adjusted Life Years)	Malaysia	Female	60+ years	Edentulism	Rate	1990	949.67	1356.35	628.14
51	All Population	DALYs (Disability-Adjusted Life Years)	Thailand	Female	60+ years	Edentulism	Rate	1992	653.99	946.77	421.65
52	All Population	DALYs (Disability-Adjusted Life Years)	Brunei Darussalam	Female	60+ years	Edentulism	Rate	1994	283.67	415.73	185.36
53	All Population	DALYs (Disability-Adjusted Life Years)	Thailand	Female	60+ years	Edentulism	Rate	1993	647.84	935.22	423.42
54	All Population	DALYs (Disability-Adjusted Life Years)	Lao People's Democratic Republic	Female	60+ years	Edentulism	Rate	1993	495.17	720.60	315.46
55	All Population	DALYs (Disability-Adjusted Life Years)	Cambodia	Female	60+ years	Edentulism	Rate	1999	730.12	1037.14	466.98
56	All Population	DALYs (Disability-Adjusted Life Years)	Thailand	Female	60+ years	Edentulism	Rate	1995	639.50	923.95	417.81
57	All Population	DALYs (Disability-Adjusted Life Years)	Lao People's Democratic Republic	Female	60+ years	Edentulism	Rate	1995	489.69	711.67	315.95
58	All Population	DALYs (Disability-Adjusted Life Years)	Philippines	Female	60+ years	Edentulism	Rate	1994	944.12	1340.89	605.37
59	All Population	DALYs (Disability-Adjusted Life Years)	Singapore	Female	60+ years	Edentulism	Rate	1996	322.90	469.00	207.78
60	All Population	DALYs (Disability-Adjusted Life Years)	Viet Nam	Female	60+ years	Edentulism	Rate	1996	671.33	960.18	432.80
61	All Population	DALYs (Disability-Adjusted Life	Lao People's	Female	60+ years	Edentulism	Rate	1996	487.53	707.52	311.81

		Years)	Democratic Republic								
62	All Population	DALYs (Disability-Adjusted Life Years)	China	Female	60+ years	Edentulism	Rate	1995	568.69	821.93	365.01
63	All Population	DALYs (Disability-Adjusted Life Years)	Singapore	Female	60+ years	Edentulism	Rate	1995	324.87	472.65	213.71
64	All Population	DALYs (Disability-Adjusted Life Years)	Malaysia	Female	60+ years	Edentulism	Rate	1994	923.80	1325.78	600.88
65	All Population	DALYs (Disability-Adjusted Life Years)	Philippines	Female	60+ years	Edentulism	Rate	1996	942.54	1335.90	604.68
66	All Population	DALYs (Disability-Adjusted Life Years)	Thailand	Female	60+ years	Edentulism	Rate	1996	636.30	918.83	407.45
67	All Population	DALYs (Disability-Adjusted Life Years)	Myanmar	Female	60+ years	Edentulism	Rate	1995	488.29	701.09	308.68
68	All Population	DALYs (Disability-Adjusted Life Years)	Association of Southeast Asian Nations	Female	60+ years	Edentulism	Rate	1993	700.91	1003.24	447.79
69	All Population	DALYs (Disability-Adjusted Life Years)	Viet Nam	Female	60+ years	Edentulism	Rate	1995	690.40	983.44	437.43
70	All Population	DALYs (Disability-Adjusted Life Years)	China	Female	60+ years	Edentulism	Rate	1994	587.97	851.32	380.08
71	All Population	DALYs (Disability-Adjusted Life Years)	Lao People's Democratic Republic	Female	60+ years	Edentulism	Rate	2000	479.85	687.02	307.31
72	All Population	DALYs (Disability-Adjusted Life Years)	Thailand	Female	60+ years	Edentulism	Rate	1994	643.95	931.65	417.27
73	All Population	DALYs (Disability-Adjusted Life Years)	Association of Southeast Asian Nations	Female	60+ years	Edentulism	Rate	1994	698.99	1001.83	446.70
74	All Population	DALYs (Disability-Adjusted Life Years)	Cambodia	Female	60+ years	Edentulism	Rate	2000	731.51	1039.05	470.97
75	All Population	DALYs (Disability-Adjusted Life Years)	Myanmar	Female	60+ years	Edentulism	Rate	1994	487.47	698.69	305.85
76	All Population	DALYs (Disability-Adjusted Life Years)	China	Female	60+ years	Edentulism	Rate	1997	506.62	738.91	324.80
77	All Population	DALYs (Disability-Adjusted Life Years)	Philippines	Female	60+ years	Edentulism	Rate	1998	941.12	1329.56	602.08
78	All Population	DALYs (Disability-Adjusted Life Years)	Lao People's Democratic Republic	Female	60+ years	Edentulism	Rate	1997	486.08	694.34	312.92
79	All Population	DALYs (Disability-Adjusted Life Years)	Philippines	Female	60+ years	Edentulism	Rate	1995	943.32	1340.08	604.23
80	All Population	DALYs (Disability-Adjusted Life Years)	Indonesia	Female	60+ years	Edentulism	Rate	1997	693.40	999.04	444.46

81	All Population	DALYs (Disability-Adjusted Life Years)	Philippines	Female	60+ years	Edentulism	Rate	1997	941.68	1334.05	603.52
82	All Population	DALYs (Disability-Adjusted Life Years)	Cambodia	Female	60+ years	Edentulism	Rate	1998	729.06	1041.12	465.25
83	All Population	DALYs (Disability-Adjusted Life Years)	Cambodia	Female	60+ years	Edentulism	Rate	2001	732.59	1053.88	469.99
84	All Population	DALYs (Disability-Adjusted Life Years)	Lao People's Democratic Republic	Female	60+ years	Edentulism	Rate	1999	481.98	695.32	310.01
85	All Population	DALYs (Disability-Adjusted Life Years)	Cambodia	Female	60+ years	Edentulism	Rate	2002	730.94	1046.96	466.95
86	All Population	DALYs (Disability-Adjusted Life Years)	Philippines	Female	60+ years	Edentulism	Rate	2000	941.43	1326.87	604.02
87	All Population	DALYs (Disability-Adjusted Life Years)	Malaysia	Female	60+ years	Edentulism	Rate	1995	923.17	1317.17	599.42
88	All Population	DALYs (Disability-Adjusted Life Years)	Viet Nam	Female	60+ years	Edentulism	Rate	1993	699.42	1009.94	443.94
89	All Population	DALYs (Disability-Adjusted Life Years)	Malaysia	Female	60+ years	Edentulism	Rate	1992	930.80	1325.22	608.97
90	All Population	DALYs (Disability-Adjusted Life Years)	Singapore	Female	60+ years	Edentulism	Rate	1999	325.49	474.09	214.22
91	All Population	DALYs (Disability-Adjusted Life Years)	Brunei Darussalam	Female	60+ years	Edentulism	Rate	1999	275.74	397.66	178.59
92	All Population	DALYs (Disability-Adjusted Life Years)	Brunei Darussalam	Female	60+ years	Edentulism	Rate	1998	279.57	411.52	181.70
93	All Population	DALYs (Disability-Adjusted Life Years)	Indonesia	Female	60+ years	Edentulism	Rate	1998	693.24	1000.83	447.53
94	All Population	DALYs (Disability-Adjusted Life Years)	Indonesia	Female	60+ years	Edentulism	Rate	1996	693.96	996.46	441.80
95	All Population	DALYs (Disability-Adjusted Life Years)	Cambodia	Female	60+ years	Edentulism	Rate	2004	728.48	1050.52	466.28
96	All Population	DALYs (Disability-Adjusted Life Years)	Lao People's Democratic Republic	Female	60+ years	Edentulism	Rate	1998	484.73	695.83	312.48
97	All Population	DALYs (Disability-Adjusted Life Years)	Thailand	Female	60+ years	Edentulism	Rate	1998	632.32	902.32	405.01
98	All Population	DALYs (Disability-Adjusted Life Years)	Cambodia	Female	60+ years	Edentulism	Rate	2005	726.79	1052.35	465.54
99	All Population	DALYs (Disability-Adjusted Life Years)	Singapore	Female	60+ years	Edentulism	Rate	2001	322.68	471.74	207.91
100	All Population	DALYs (Disability-Adjusted Life Years)	Indonesia	Female	60+ years	Edentulism	Rate	2000	696.39	1004.88	452.29
101	All Population	DALYs (Disability-Adjusted Life Years)	Malaysia	Female	60+ years	Edentulism	Rate	1999	1470.56	2012.00	1010.60

102	All Population	DALYs (Disability-Adjusted Life Years)	Philippines	Female	60+ years	Edentulism	Rate	2001	939.14	1327.25	601.30
103	All Population	DALYs (Disability-Adjusted Life Years)	Thailand	Female	60+ years	Edentulism	Rate	1997	633.95	907.38	406.54
104	All Population	DALYs (Disability-Adjusted Life Years)	Philippines	Female	60+ years	Edentulism	Rate	2002	932.52	1323.84	598.38
105	All Population	DALYs (Disability-Adjusted Life Years)	Philippines	Female	60+ years	Edentulism	Rate	1999	941.02	1328.98	603.25
106	All Population	DALYs (Disability-Adjusted Life Years)	China	Female	60+ years	Edentulism	Rate	1999	447.24	650.99	288.81
107	All Population	DALYs (Disability-Adjusted Life Years)	Thailand	Female	60+ years	Edentulism	Rate	2000	631.15	905.42	403.76
108	All Population	DALYs (Disability-Adjusted Life Years)	Philippines	Female	60+ years	Edentulism	Rate	2004	917.87	1302.95	588.30
109	All Population	DALYs (Disability-Adjusted Life Years)	Association of Southeast Asian Nations	Female	60+ years	Edentulism	Rate	1995	697.62	1000.48	445.57
110	All Population	DALYs (Disability-Adjusted Life Years)	Thailand	Female	60+ years	Edentulism	Rate	2004	640.31	918.66	411.62
111	All Population	DALYs (Disability-Adjusted Life Years)	Singapore	Female	60+ years	Edentulism	Rate	2002	322.80	468.96	210.76
112	All Population	DALYs (Disability-Adjusted Life Years)	Lao People's Democratic Republic	Female	60+ years	Edentulism	Rate	2001	460.25	654.95	299.66
113	All Population	DALYs (Disability-Adjusted Life Years)	Lao People's Democratic Republic	Female	60+ years	Edentulism	Rate	2002	419.86	591.88	276.73
114	All Population	DALYs (Disability-Adjusted Life Years)	Philippines	Female	60+ years	Edentulism	Rate	2005	917.05	1299.14	586.79
115	All Population	DALYs (Disability-Adjusted Life Years)	Cambodia	Female	60+ years	Edentulism	Rate	2006	723.36	1045.18	464.57
116	All Population	DALYs (Disability-Adjusted Life Years)	Cambodia	Female	60+ years	Edentulism	Rate	2007	718.45	1033.79	459.92
117	All Population	DALYs (Disability-Adjusted Life Years)	Brunei Darussalam	Female	60+ years	Edentulism	Rate	1995	286.76	423.48	188.79
118	All Population	DALYs (Disability-Adjusted Life Years)	Thailand	Female	60+ years	Edentulism	Rate	2003	637.80	918.20	409.77
119	All Population	DALYs (Disability-Adjusted Life Years)	China	Female	60+ years	Edentulism	Rate	2000	438.00	638.17	283.05
120	All Population	DALYs (Disability-Adjusted Life Years)	China	Female	60+ years	Edentulism	Rate	1996	541.50	787.21	348.38
121	All Population	DALYs (Disability-Adjusted Life Years)	Myanmar	Female	60+ years	Edentulism	Rate	2000	487.80	708.38	316.68
122	All Population	DALYs (Disability-Adjusted Life	Brunei	Female	60+ years	Edentulism	Rate	1997	282.59	408.61	185.51

		Years)	Darussalam								
123	All Population	DALYs (Disability-Adjusted Life Years)	Lao People's Democratic Republic	Female	60+ years	Edentulism	Rate	2004	337.56	474.04	222.98
124	All Population	DALYs (Disability-Adjusted Life Years)	Lao People's Democratic Republic	Female	60+ years	Edentulism	Rate	2005	323.20	457.20	210.52
125	All Population	DALYs (Disability-Adjusted Life Years)	Brunei Darussalam	Female	60+ years	Edentulism	Rate	2002	271.22	386.87	176.66
126	All Population	DALYs (Disability-Adjusted Life Years)	Thailand	Female	60+ years	Edentulism	Rate	1999	631.27	914.02	405.21
127	All Population	DALYs (Disability-Adjusted Life Years)	Malaysia	Female	60+ years	Edentulism	Rate	1997	1134.73	1571.92	771.29
128	All Population	DALYs (Disability-Adjusted Life Years)	Association of Southeast Asian Nations	Female	60+ years	Edentulism	Rate	1998	691.86	977.44	449.54
129	All Population	DALYs (Disability-Adjusted Life Years)	Brunei Darussalam	Female	60+ years	Edentulism	Rate	2001	274.99	398.12	178.44
130	All Population	DALYs (Disability-Adjusted Life Years)	Association of Southeast Asian Nations	Female	60+ years	Edentulism	Rate	1999	691.68	974.04	451.57
131	All Population	DALYs (Disability-Adjusted Life Years)	Brunei Darussalam	Female	60+ years	Edentulism	Rate	1996	282.74	414.48	184.30
132	All Population	DALYs (Disability-Adjusted Life Years)	Thailand	Female	60+ years	Edentulism	Rate	2002	634.91	908.45	407.33
133	All Population	DALYs (Disability-Adjusted Life Years)	Indonesia	Female	60+ years	Edentulism	Rate	2002	711.34	999.93	476.96
134	All Population	DALYs (Disability-Adjusted Life Years)	Viet Nam	Female	60+ years	Edentulism	Rate	1992	703.52	1006.74	452.71
135	All Population	DALYs (Disability-Adjusted Life Years)	Philippines	Female	60+ years	Edentulism	Rate	2003	924.05	1311.81	594.20
136	All Population	DALYs (Disability-Adjusted Life Years)	Thailand	Female	60+ years	Edentulism	Rate	2001	633.71	916.73	410.33
137	All Population	DALYs (Disability-Adjusted Life Years)	Lao People's Democratic Republic	Female	60+ years	Edentulism	Rate	2006	338.70	473.42	223.08
138	All Population	DALYs (Disability-Adjusted Life Years)	Cambodia	Female	60+ years	Edentulism	Rate	2008	711.72	1026.32	461.70
139	All Population	DALYs (Disability-Adjusted Life Years)	Lao People's Democratic Republic	Female	60+ years	Edentulism	Rate	2008	414.23	587.53	271.02
140	All Population	DALYs (Disability-Adjusted Life Years)	China	Female	60+ years	Edentulism	Rate	2001	448.23	653.74	289.58
141	All Population	DALYs (Disability-Adjusted Life	Thailand	Female	60+ years	Edentulism	Rate	2005	641.96	919.69	414.76

		Years)									
142	All Population	DALYs (Disability-Adjusted Life Years)	Viet Nam	Female	60+ years	Edentulism	Rate	1997	632.74	901.31	414.60
143	All Population	DALYs (Disability-Adjusted Life Years)	Lao People's Democratic Republic	Female	60+ years	Edentulism	Rate	2007	373.23	524.18	245.82
144	All Population	DALYs (Disability-Adjusted Life Years)	Singapore	Female	60+ years	Edentulism	Rate	2004	326.87	470.35	209.68
145	All Population	DALYs (Disability-Adjusted Life Years)	China	Female	60+ years	Edentulism	Rate	2002	471.10	684.36	306.47
146	All Population	DALYs (Disability-Adjusted Life Years)	Cambodia	Female	60+ years	Edentulism	Rate	2003	730.21	1056.89	465.18
147	All Population	DALYs (Disability-Adjusted Life Years)	Brunei Darussalam	Female	60+ years	Edentulism	Rate	2000	278.87	402.28	181.69
148	All Population	DALYs (Disability-Adjusted Life Years)	Association of Southeast Asian Nations	Female	60+ years	Edentulism	Rate	2000	692.38	977.39	454.94
149	All Population	DALYs (Disability-Adjusted Life Years)	Myanmar	Female	60+ years	Edentulism	Rate	2001	465.48	667.55	305.17
150	All Population	DALYs (Disability-Adjusted Life Years)	Cambodia	Female	60+ years	Edentulism	Rate	2009	704.55	1020.52	456.04
151	All Population	DALYs (Disability-Adjusted Life Years)	Viet Nam	Female	60+ years	Edentulism	Rate	2002	537.79	747.79	370.84
152	All Population	DALYs (Disability-Adjusted Life Years)	Malaysia	Female	60+ years	Edentulism	Rate	1996	983.41	1365.19	662.45
153	All Population	DALYs (Disability-Adjusted Life Years)	Lao People's Democratic Republic	Female	60+ years	Edentulism	Rate	2003	374.16	520.68	246.99
154	All Population	DALYs (Disability-Adjusted Life Years)	Myanmar	Female	60+ years	Edentulism	Rate	1997	489.00	694.46	315.52
155	All Population	DALYs (Disability-Adjusted Life Years)	Association of Southeast Asian Nations	Female	60+ years	Edentulism	Rate	2001	693.11	969.98	459.74
156	All Population	DALYs (Disability-Adjusted Life Years)	Malaysia	Female	60+ years	Edentulism	Rate	1998	1316.14	1817.03	910.80
157	All Population	DALYs (Disability-Adjusted Life Years)	Singapore	Female	60+ years	Edentulism	Rate	2003	326.31	471.13	213.69
158	All Population	DALYs (Disability-Adjusted Life Years)	Viet Nam	Female	60+ years	Edentulism	Rate	1999	551.30	775.71	365.76
159	All Population	DALYs (Disability-Adjusted Life Years)	Viet Nam	Female	60+ years	Edentulism	Rate	2004	545.21	759.06	375.44
160	All Population	DALYs (Disability-Adjusted Life Years)	Brunei Darussalam	Female	60+ years	Edentulism	Rate	2004	271.20	389.74	177.41
161	All Population	DALYs (Disability-Adjusted Life	Association of	Female	60+ years	Edentulism	Rate	1996	695.34	993.81	445.65

		Years)	Southeast Asian Nations								
162	All Population	DALYs (Disability-Adjusted Life Years)	Myanmar	Female	60+ years	Edentulism	Rate	2002	415.06	594.39	271.25
163	All Population	DALYs (Disability-Adjusted Life Years)	Malaysia	Female	60+ years	Edentulism	Rate	2000	1537.15	2101.36	1034.18
164	All Population	DALYs (Disability-Adjusted Life Years)	Indonesia	Female	60+ years	Edentulism	Rate	2004	730.01	999.73	500.80
165	All Population	DALYs (Disability-Adjusted Life Years)	Viet Nam	Female	60+ years	Edentulism	Rate	2005	548.32	761.24	375.77
166	All Population	DALYs (Disability-Adjusted Life Years)	Lao People's Democratic Republic	Female	60+ years	Edentulism	Rate	2009	447.37	643.62	292.94
167	All Population	DALYs (Disability-Adjusted Life Years)	Association of Southeast Asian Nations	Female	60+ years	Edentulism	Rate	1997	693.39	984.26	447.42
168	All Population	DALYs (Disability-Adjusted Life Years)	Viet Nam	Female	60+ years	Edentulism	Rate	2003	541.83	745.30	373.62
169	All Population	DALYs (Disability-Adjusted Life Years)	Viet Nam	Female	60+ years	Edentulism	Rate	1998	587.89	828.31	383.19
170	All Population	DALYs (Disability-Adjusted Life Years)	Singapore	Female	60+ years	Edentulism	Rate	1997	323.38	473.74	209.61
171	All Population	DALYs (Disability-Adjusted Life Years)	Indonesia	Female	60+ years	Edentulism	Rate	2005	733.70	994.90	499.96
172	All Population	DALYs (Disability-Adjusted Life Years)	Myanmar	Female	60+ years	Edentulism	Rate	1996	488.17	698.03	311.70
173	All Population	DALYs (Disability-Adjusted Life Years)	China	Female	60+ years	Edentulism	Rate	2004	526.55	764.19	345.13
174	All Population	DALYs (Disability-Adjusted Life Years)	Brunei Darussalam	Female	60+ years	Edentulism	Rate	2003	271.07	389.29	177.13
175	All Population	DALYs (Disability-Adjusted Life Years)	Singapore	Female	60+ years	Edentulism	Rate	2005	331.49	474.18	215.02
176	All Population	DALYs (Disability-Adjusted Life Years)	Malaysia	Female	60+ years	Edentulism	Rate	2002	1535.90	2097.90	1058.09
177	All Population	DALYs (Disability-Adjusted Life Years)	Cambodia	Female	60+ years	Edentulism	Rate	2010	698.45	1012.78	441.21
178	All Population	DALYs (Disability-Adjusted Life Years)	Cambodia	Female	60+ years	Edentulism	Rate	2011	692.70	1001.03	448.67
179	All Population	DALYs (Disability-Adjusted Life Years)	China	Female	60+ years	Edentulism	Rate	2005	544.61	789.83	356.46
180	All Population	DALYs (Disability-Adjusted Life Years)	China	Female	60+ years	Edentulism	Rate	1998	472.51	688.25	302.88
181	All Population	DALYs (Disability-Adjusted Life Years)	Association of Southeast	Female	60+ years	Edentulism	Rate	2003	690.83	949.71	464.59

			Asian Nations								
182	All Population	DALYs (Disability-Adjusted Life Years)	Indonesia	Female	60+ years	Edentulism	Rate	2003	721.64	998.92	493.79
183	All Population	DALYs (Disability-Adjusted Life Years)	Singapore	Female	60+ years	Edentulism	Rate	2000	324.45	473.72	213.84
184	All Population	DALYs (Disability-Adjusted Life Years)	Lao People's Democratic Republic	Female	60+ years	Edentulism	Rate	2010	456.47	656.67	302.57
185	All Population	DALYs (Disability-Adjusted Life Years)	Lao People's Democratic Republic	Female	60+ years	Edentulism	Rate	2011	454.28	652.73	298.47
186	All Population	DALYs (Disability-Adjusted Life Years)	Association of Southeast Asian Nations	Female	60+ years	Edentulism	Rate	2004	689.55	944.40	468.61
187	All Population	DALYs (Disability-Adjusted Life Years)	Singapore	Female	60+ years	Edentulism	Rate	2006	332.55	480.60	211.77
188	All Population	DALYs (Disability-Adjusted Life Years)	Philippines	Female	60+ years	Edentulism	Rate	2006	924.73	1309.43	607.36
189	All Population	DALYs (Disability-Adjusted Life Years)	Singapore	Female	60+ years	Edentulism	Rate	2007	326.05	472.71	210.18
190	All Population	DALYs (Disability-Adjusted Life Years)	Thailand	Female	60+ years	Edentulism	Rate	2006	639.71	920.04	413.38
191	All Population	DALYs (Disability-Adjusted Life Years)	Malaysia	Female	60+ years	Edentulism	Rate	2004	1496.45	2045.45	1021.39
192	All Population	DALYs (Disability-Adjusted Life Years)	Brunei Darussalam	Female	60+ years	Edentulism	Rate	2006	278.62	403.49	181.72
193	All Population	DALYs (Disability-Adjusted Life Years)	Brunei Darussalam	Female	60+ years	Edentulism	Rate	2005	276.69	401.57	180.93
194	All Population	DALYs (Disability-Adjusted Life Years)	Brunei Darussalam	Female	60+ years	Edentulism	Rate	2007	276.00	397.69	178.30
195	All Population	DALYs (Disability-Adjusted Life Years)	China	Female	60+ years	Edentulism	Rate	2003	499.63	726.10	326.38
196	All Population	DALYs (Disability-Adjusted Life Years)	Cambodia	Female	60+ years	Edentulism	Rate	2012	687.57	992.17	437.20
197	All Population	DALYs (Disability-Adjusted Life Years)	Viet Nam	Female	60+ years	Edentulism	Rate	2000	534.97	747.00	355.02
198	All Population	DALYs (Disability-Adjusted Life Years)	Indonesia	Female	60+ years	Edentulism	Rate	1999	694.27	1001.73	449.35
199	All Population	DALYs (Disability-Adjusted Life Years)	Lao People's Democratic Republic	Female	60+ years	Edentulism	Rate	2012	447.78	642.89	292.82
200	All Population	DALYs (Disability-Adjusted Life Years)	Myanmar	Female	60+ years	Edentulism	Rate	1998	489.01	697.37	312.00
201	All Population	DALYs (Disability-Adjusted Life	Singapore	Female	60+ years	Edentulism	Rate	2009	317.44	462.59	204.11

		Years)									
202	All Population	DALYs (Disability-Adjusted Life Years)	Indonesia	Female	60+ years	Edentulism	Rate	2006	734.26	998.75	496.60
203	All Population	DALYs (Disability-Adjusted Life Years)	Cambodia	Female	60+ years	Edentulism	Rate	2013	683.73	982.75	439.78
204	All Population	DALYs (Disability-Adjusted Life Years)	Lao People's Democratic Republic	Female	60+ years	Edentulism	Rate	2013	439.04	631.51	283.39
205	All Population	DALYs (Disability-Adjusted Life Years)	Myanmar	Female	60+ years	Edentulism	Rate	2003	355.90	510.70	233.47
206	All Population	DALYs (Disability-Adjusted Life Years)	Malaysia	Female	60+ years	Edentulism	Rate	2007	1229.45	1673.19	852.28
207	All Population	DALYs (Disability-Adjusted Life Years)	Malaysia	Female	60+ years	Edentulism	Rate	2005	1464.86	1992.14	1000.76
208	All Population	DALYs (Disability-Adjusted Life Years)	China	Female	60+ years	Edentulism	Rate	2007	561.18	812.21	367.88
209	All Population	DALYs (Disability-Adjusted Life Years)	Association of Southeast Asian Nations	Female	60+ years	Edentulism	Rate	2007	689.70	939.90	474.57
210	All Population	DALYs (Disability-Adjusted Life Years)	Cambodia	Female	60+ years	Edentulism	Rate	2014	677.78	969.33	444.69
211	All Population	DALYs (Disability-Adjusted Life Years)	Indonesia	Female	60+ years	Edentulism	Rate	2001	701.97	999.79	463.62
212	All Population	DALYs (Disability-Adjusted Life Years)	Singapore	Female	60+ years	Edentulism	Rate	1998	326.44	467.89	216.41
213	All Population	DALYs (Disability-Adjusted Life Years)	Indonesia	Female	60+ years	Edentulism	Rate	2009	731.19	996.33	485.02
214	All Population	DALYs (Disability-Adjusted Life Years)	Singapore	Female	60+ years	Edentulism	Rate	2008	320.07	462.45	206.71
215	All Population	DALYs (Disability-Adjusted Life Years)	Viet Nam	Female	60+ years	Edentulism	Rate	2006	548.34	763.62	379.59
216	All Population	DALYs (Disability-Adjusted Life Years)	Viet Nam	Female	60+ years	Edentulism	Rate	2010	545.43	755.71	375.36
217	All Population	DALYs (Disability-Adjusted Life Years)	Lao People's Democratic Republic	Female	60+ years	Edentulism	Rate	2014	431.01	615.32	281.86
218	All Population	DALYs (Disability-Adjusted Life Years)	Philippines	Female	60+ years	Edentulism	Rate	2007	940.48	1312.37	622.89
219	All Population	DALYs (Disability-Adjusted Life Years)	China	Female	60+ years	Edentulism	Rate	2008	568.21	823.82	373.98
220	All Population	DALYs (Disability-Adjusted Life Years)	Myanmar	Female	60+ years	Edentulism	Rate	2008	393.93	565.32	258.35
221	All Population	DALYs (Disability-Adjusted Life Years)	China	Female	60+ years	Edentulism	Rate	2006	553.91	802.90	363.27

222	All Population	DALYs (Disability-Adjusted Life Years)	Myanmar	Female	60+ years	Edentulism	Rate	2007	345.41	494.47	230.33
223	All Population	DALYs (Disability-Adjusted Life Years)	Myanmar	Female	60+ years	Edentulism	Rate	2004	306.11	436.14	205.88
224	All Population	DALYs (Disability-Adjusted Life Years)	Myanmar	Female	60+ years	Edentulism	Rate	2005	286.03	402.83	192.76
225	All Population	DALYs (Disability-Adjusted Life Years)	Brunei Darussalam	Female	60+ years	Edentulism	Rate	2009	271.70	397.11	177.37
226	All Population	DALYs (Disability-Adjusted Life Years)	Thailand	Female	60+ years	Edentulism	Rate	2007	636.31	919.05	406.40
227	All Population	DALYs (Disability-Adjusted Life Years)	Indonesia	Female	60+ years	Edentulism	Rate	2007	734.35	999.44	496.77
228	All Population	DALYs (Disability-Adjusted Life Years)	Indonesia	Female	60+ years	Edentulism	Rate	2010	727.65	993.83	480.42
229	All Population	DALYs (Disability-Adjusted Life Years)	Indonesia	Female	60+ years	Edentulism	Rate	2008	733.41	995.33	489.57
230	All Population	DALYs (Disability-Adjusted Life Years)	Association of Southeast Asian Nations	Female	60+ years	Edentulism	Rate	2002	692.19	962.68	461.25
231	All Population	DALYs (Disability-Adjusted Life Years)	Association of Southeast Asian Nations	Female	60+ years	Edentulism	Rate	2006	690.13	948.91	474.78
232	All Population	DALYs (Disability-Adjusted Life Years)	Myanmar	Female	60+ years	Edentulism	Rate	2006	303.37	434.27	204.06
233	All Population	DALYs (Disability-Adjusted Life Years)	Brunei Darussalam	Female	60+ years	Edentulism	Rate	2008	275.53	399.94	179.94
234	All Population	DALYs (Disability-Adjusted Life Years)	Singapore	Female	60+ years	Edentulism	Rate	2010	315.52	454.34	201.03
235	All Population	DALYs (Disability-Adjusted Life Years)	Association of Southeast Asian Nations	Female	60+ years	Edentulism	Rate	2008	688.69	936.37	473.98
236	All Population	DALYs (Disability-Adjusted Life Years)	China	Female	60+ years	Edentulism	Rate	2009	568.16	825.55	374.45
237	All Population	DALYs (Disability-Adjusted Life Years)	Cambodia	Female	60+ years	Edentulism	Rate	2015	673.77	961.08	440.43
238	All Population	DALYs (Disability-Adjusted Life Years)	Association of Southeast Asian Nations	Female	60+ years	Edentulism	Rate	2009	686.46	929.35	475.14
239	All Population	DALYs (Disability-Adjusted Life Years)	Singapore	Female	60+ years	Edentulism	Rate	2011	313.29	450.89	200.75
240	All Population	DALYs (Disability-Adjusted Life Years)	Singapore	Female	60+ years	Edentulism	Rate	2012	311.50	456.92	203.06
241	All Population	DALYs (Disability-Adjusted Life Years)	Lao People's Democratic	Female	60+ years	Edentulism	Rate	2015	426.38	609.95	270.87

			Republic								
242	All Population	DALYs (Disability-Adjusted Life Years)	Myanmar	Female	60+ years	Edentulism	Rate	2009	432.89	629.04	284.11
243	All Population	DALYs (Disability-Adjusted Life Years)	Myanmar	Female	60+ years	Edentulism	Rate	2010	449.75	653.71	298.45
244	All Population	DALYs (Disability-Adjusted Life Years)	Malaysia	Female	60+ years	Edentulism	Rate	2006	1374.60	1877.86	953.49
245	All Population	DALYs (Disability-Adjusted Life Years)	Malaysia	Female	60+ years	Edentulism	Rate	2003	1520.37	2084.49	1037.06
246	All Population	DALYs (Disability-Adjusted Life Years)	China	Female	60+ years	Edentulism	Rate	2010	569.21	826.60	375.83
247	All Population	DALYs (Disability-Adjusted Life Years)	Association of Southeast Asian Nations	Female	60+ years	Edentulism	Rate	2005	689.74	941.31	474.03
248	All Population	DALYs (Disability-Adjusted Life Years)	Brunei Darussalam	Female	60+ years	Edentulism	Rate	2010	270.88	395.21	174.41
249	All Population	DALYs (Disability-Adjusted Life Years)	Myanmar	Female	60+ years	Edentulism	Rate	1999	489.86	703.93	317.30
250	All Population	DALYs (Disability-Adjusted Life Years)	Viet Nam	Female	60+ years	Edentulism	Rate	2012	565.95	788.27	388.20
251	All Population	DALYs (Disability-Adjusted Life Years)	Philippines	Female	60+ years	Edentulism	Rate	2008	957.46	1317.55	642.11
252	All Population	DALYs (Disability-Adjusted Life Years)	Viet Nam	Female	60+ years	Edentulism	Rate	2011	549.13	758.48	375.88
253	All Population	DALYs (Disability-Adjusted Life Years)	Viet Nam	Female	60+ years	Edentulism	Rate	2007	547.04	762.57	376.47
254	All Population	DALYs (Disability-Adjusted Life Years)	Malaysia	Female	60+ years	Edentulism	Rate	2009	919.33	1279.27	635.07
255	All Population	DALYs (Disability-Adjusted Life Years)	Brunei Darussalam	Female	60+ years	Edentulism	Rate	2011	268.62	391.61	173.44
256	All Population	DALYs (Disability-Adjusted Life Years)	China	Female	60+ years	Edentulism	Rate	2011	553.37	799.33	366.55
257	All Population	DALYs (Disability-Adjusted Life Years)	Thailand	Female	60+ years	Edentulism	Rate	2008	631.47	914.88	410.33
258	All Population	DALYs (Disability-Adjusted Life Years)	Malaysia	Female	60+ years	Edentulism	Rate	2001	1540.10	2094.74	1059.62
259	All Population	DALYs (Disability-Adjusted Life Years)	Indonesia	Female	60+ years	Edentulism	Rate	2012	717.51	976.41	472.01
260	All Population	DALYs (Disability-Adjusted Life Years)	Viet Nam	Female	60+ years	Edentulism	Rate	2013	586.50	819.24	404.55
261	All Population	DALYs (Disability-Adjusted Life Years)	Indonesia	Female	60+ years	Edentulism	Rate	2013	710.80	967.81	463.20
262	All Population	DALYs (Disability-Adjusted Life Years)	Myanmar	Female	60+ years	Edentulism	Rate	2011	445.50	646.36	289.49

263	All Population	DALYs (Disability-Adjusted Life Years)	Association of Southeast Asian Nations	Female	60+ years	Edentulism	Rate	2010	685.47	924.99	476.46
264	All Population	DALYs (Disability-Adjusted Life Years)	Singapore	Female	60+ years	Edentulism	Rate	2013	309.58	448.57	201.72
265	All Population	DALYs (Disability-Adjusted Life Years)	Cambodia	Female	60+ years	Edentulism	Rate	2016	668.96	965.89	434.79
266	All Population	DALYs (Disability-Adjusted Life Years)	Malaysia	Female	60+ years	Edentulism	Rate	2008	1061.73	1471.14	737.62
267	All Population	DALYs (Disability-Adjusted Life Years)	Association of Southeast Asian Nations	Female	60+ years	Edentulism	Rate	2011	683.27	927.11	474.30
268	All Population	DALYs (Disability-Adjusted Life Years)	China	Female	60+ years	Edentulism	Rate	2012	519.30	744.89	345.97
269	All Population	DALYs (Disability-Adjusted Life Years)	Lao People's Democratic Republic	Female	60+ years	Edentulism	Rate	2016	421.09	600.98	269.44
270	All Population	DALYs (Disability-Adjusted Life Years)	Malaysia	Female	60+ years	Edentulism	Rate	2010	852.24	1181.76	583.85
271	All Population	DALYs (Disability-Adjusted Life Years)	Viet Nam	Female	60+ years	Edentulism	Rate	2009	543.36	759.55	371.28
272	All Population	DALYs (Disability-Adjusted Life Years)	Singapore	Female	60+ years	Edentulism	Rate	2014	309.29	450.49	199.72
273	All Population	DALYs (Disability-Adjusted Life Years)	Brunei Darussalam	Female	60+ years	Edentulism	Rate	2012	265.71	389.12	169.92
274	All Population	DALYs (Disability-Adjusted Life Years)	Myanmar	Female	60+ years	Edentulism	Rate	2012	437.95	627.42	281.37
275	All Population	DALYs (Disability-Adjusted Life Years)	Indonesia	Female	60+ years	Edentulism	Rate	2014	703.99	959.98	458.37
276	All Population	DALYs (Disability-Adjusted Life Years)	Viet Nam	Female	60+ years	Edentulism	Rate	2014	603.43	850.62	406.35
277	All Population	DALYs (Disability-Adjusted Life Years)	Malaysia	Female	60+ years	Edentulism	Rate	2012	841.95	1172.32	565.57
278	All Population	DALYs (Disability-Adjusted Life Years)	Viet Nam	Female	60+ years	Edentulism	Rate	2001	534.91	740.83	360.46
279	All Population	DALYs (Disability-Adjusted Life Years)	Indonesia	Female	60+ years	Edentulism	Rate	2011	723.37	987.86	476.14
280	All Population	DALYs (Disability-Adjusted Life Years)	Malaysia	Female	60+ years	Edentulism	Rate	2013	838.63	1161.41	554.53
281	All Population	DALYs (Disability-Adjusted Life Years)	Viet Nam	Female	60+ years	Edentulism	Rate	2008	545.34	752.55	373.08
282	All Population	DALYs (Disability-Adjusted Life Years)	Singapore	Female	60+ years	Edentulism	Rate	2015	307.78	451.84	198.89
283	All Population	DALYs (Disability-Adjusted Life	Cambodia	Female	60+ years	Edentulism	Rate	2017	663.07	941.47	426.79

		Years)									
284	All Population	DALYs (Disability-Adjusted Life Years)	Malaysia	Female	60+ years	Edentulism	Rate	2014	838.57	1186.22	540.27
285	All Population	DALYs (Disability-Adjusted Life Years)	Malaysia	Female	60+ years	Edentulism	Rate	2015	838.05	1205.80	536.17
286	All Population	DALYs (Disability-Adjusted Life Years)	Lao People's Democratic Republic	Female	60+ years	Edentulism	Rate	2017	414.94	595.48	265.50
287	All Population	DALYs (Disability-Adjusted Life Years)	Association of Southeast Asian Nations	Female	60+ years	Edentulism	Rate	2012	682.52	925.66	473.13
288	All Population	DALYs (Disability-Adjusted Life Years)	Indonesia	Female	60+ years	Edentulism	Rate	2015	697.52	948.67	453.26
289	All Population	DALYs (Disability-Adjusted Life Years)	Malaysia	Female	60+ years	Edentulism	Rate	2011	846.81	1173.81	573.70
290	All Population	DALYs (Disability-Adjusted Life Years)	China	Female	60+ years	Edentulism	Rate	2013	478.38	685.51	318.38
291	All Population	DALYs (Disability-Adjusted Life Years)	Viet Nam	Female	60+ years	Edentulism	Rate	2015	604.52	870.57	397.03
292	All Population	DALYs (Disability-Adjusted Life Years)	Brunei Darussalam	Female	60+ years	Edentulism	Rate	2013	264.57	388.19	170.77
293	All Population	DALYs (Disability-Adjusted Life Years)	Indonesia	Female	60+ years	Edentulism	Rate	2016	691.51	944.41	447.14
294	All Population	DALYs (Disability-Adjusted Life Years)	Singapore	Female	60+ years	Edentulism	Rate	2016	304.64	447.09	195.50
295	All Population	DALYs (Disability-Adjusted Life Years)	Philippines	Female	60+ years	Edentulism	Rate	2009	970.93	1327.24	659.41
296	All Population	DALYs (Disability-Adjusted Life Years)	Thailand	Female	60+ years	Edentulism	Rate	2009	625.27	902.52	403.95
297	All Population	DALYs (Disability-Adjusted Life Years)	Malaysia	Female	60+ years	Edentulism	Rate	2016	835.76	1188.83	536.38
298	All Population	DALYs (Disability-Adjusted Life Years)	Cambodia	Female	60+ years	Edentulism	Rate	2018	658.99	945.56	419.19
299	All Population	DALYs (Disability-Adjusted Life Years)	Lao People's Democratic Republic	Female	60+ years	Edentulism	Rate	2018	409.72	591.84	262.40
300	All Population	DALYs (Disability-Adjusted Life Years)	Myanmar	Female	60+ years	Edentulism	Rate	2013	429.05	619.51	276.79
301	All Population	DALYs (Disability-Adjusted Life Years)	Association of Southeast Asian Nations	Female	60+ years	Edentulism	Rate	2013	682.45	929.55	473.14
302	All Population	DALYs (Disability-Adjusted Life Years)	Viet Nam	Female	60+ years	Edentulism	Rate	2016	598.91	867.85	402.61
303	All Population	DALYs (Disability-Adjusted Life	China	Female	60+ years	Edentulism	Rate	2014	445.51	633.51	296.44

		Years)									
304	All Population	DALYs (Disability-Adjusted Life Years)	Myanmar	Female	60+ years	Edentulism	Rate	2014	419.88	600.43	270.34
305	All Population	DALYs (Disability-Adjusted Life Years)	Indonesia	Female	60+ years	Edentulism	Rate	2017	685.58	929.05	444.50
306	All Population	DALYs (Disability-Adjusted Life Years)	Singapore	Female	60+ years	Edentulism	Rate	2017	304.75	445.02	196.09
307	All Population	DALYs (Disability-Adjusted Life Years)	Philippines	Female	60+ years	Edentulism	Rate	2010	978.04	1325.13	673.94
308	All Population	DALYs (Disability-Adjusted Life Years)	Brunei Darussalam	Female	60+ years	Edentulism	Rate	2014	260.69	380.86	168.48
309	All Population	DALYs (Disability-Adjusted Life Years)	Malaysia	Female	60+ years	Edentulism	Rate	2017	828.92	1190.29	529.82
310	All Population	DALYs (Disability-Adjusted Life Years)	Thailand	Female	60+ years	Edentulism	Rate	2010	623.00	904.38	399.61
311	All Population	DALYs (Disability-Adjusted Life Years)	Viet Nam	Female	60+ years	Edentulism	Rate	2017	588.95	852.63	387.64
312	All Population	DALYs (Disability-Adjusted Life Years)	Viet Nam	Female	60+ years	Edentulism	Rate	2018	579.22	826.30	377.47
313	All Population	DALYs (Disability-Adjusted Life Years)	Indonesia	Female	60+ years	Edentulism	Rate	2018	680.32	924.87	439.83
314	All Population	DALYs (Disability-Adjusted Life Years)	Association of Southeast Asian Nations	Female	60+ years	Edentulism	Rate	2014	681.72	931.92	472.59
315	All Population	DALYs (Disability-Adjusted Life Years)	China	Female	60+ years	Edentulism	Rate	2015	430.79	609.82	287.63
316	All Population	DALYs (Disability-Adjusted Life Years)	Myanmar	Female	60+ years	Edentulism	Rate	2015	413.00	586.87	262.80
317	All Population	DALYs (Disability-Adjusted Life Years)	Malaysia	Female	60+ years	Edentulism	Rate	2018	822.94	1183.86	521.95
318	All Population	DALYs (Disability-Adjusted Life Years)	Singapore	Female	60+ years	Edentulism	Rate	2018	303.50	442.98	197.42
319	All Population	DALYs (Disability-Adjusted Life Years)	Brunei Darussalam	Female	60+ years	Edentulism	Rate	2015	257.81	374.42	165.21
320	All Population	DALYs (Disability-Adjusted Life Years)	Cambodia	Female	60+ years	Edentulism	Rate	2019	654.72	943.33	415.18
321	All Population	DALYs (Disability-Adjusted Life Years)	Lao People's Democratic Republic	Female	60+ years	Edentulism	Rate	2019	405.95	586.57	260.84
322	All Population	DALYs (Disability-Adjusted Life Years)	Association of Southeast Asian Nations	Female	60+ years	Edentulism	Rate	2015	677.78	927.71	466.26
323	All Population	DALYs (Disability-Adjusted Life Years)	China	Female	60+ years	Edentulism	Rate	2016	455.76	644.31	305.62

324	All Population	DALYs (Disability-Adjusted Life Years)	Myanmar	Female	60+ years	Edentulism	Rate	2016	408.25	583.02	264.06
325	All Population	DALYs (Disability-Adjusted Life Years)	Brunei Darussalam	Female	60+ years	Edentulism	Rate	2016	255.11	370.81	165.70
326	All Population	DALYs (Disability-Adjusted Life Years)	Philippines	Female	60+ years	Edentulism	Rate	2011	979.89	1325.07	681.69
327	All Population	DALYs (Disability-Adjusted Life Years)	Thailand	Female	60+ years	Edentulism	Rate	2011	619.69	898.18	400.36
328	All Population	DALYs (Disability-Adjusted Life Years)	Viet Nam	Female	60+ years	Edentulism	Rate	2019	569.45	812.08	369.81
329	All Population	DALYs (Disability-Adjusted Life Years)	Indonesia	Female	60+ years	Edentulism	Rate	2019	676.82	923.03	442.00
330	All Population	DALYs (Disability-Adjusted Life Years)	Singapore	Female	60+ years	Edentulism	Rate	2019	305.69	443.37	194.55
331	All Population	DALYs (Disability-Adjusted Life Years)	Malaysia	Female	60+ years	Edentulism	Rate	2019	820.33	1174.79	525.81
332	All Population	DALYs (Disability-Adjusted Life Years)	Cambodia	Female	60+ years	Edentulism	Rate	2020	649.18	922.38	409.44
333	All Population	DALYs (Disability-Adjusted Life Years)	Lao People's Democratic Republic	Female	60+ years	Edentulism	Rate	2020	403.45	581.76	264.79
334	All Population	DALYs (Disability-Adjusted Life Years)	Association of Southeast Asian Nations	Female	60+ years	Edentulism	Rate	2016	672.76	922.58	463.16
335	All Population	DALYs (Disability-Adjusted Life Years)	Myanmar	Female	60+ years	Edentulism	Rate	2017	403.43	586.30	260.55
336	All Population	DALYs (Disability-Adjusted Life Years)	China	Female	60+ years	Edentulism	Rate	2017	512.77	723.22	346.31
337	All Population	DALYs (Disability-Adjusted Life Years)	Brunei Darussalam	Female	60+ years	Edentulism	Rate	2017	255.94	373.07	166.66
338	All Population	DALYs (Disability-Adjusted Life Years)	Viet Nam	Female	60+ years	Edentulism	Rate	2020	562.49	822.03	365.88
339	All Population	DALYs (Disability-Adjusted Life Years)	Indonesia	Female	60+ years	Edentulism	Rate	2020	673.51	912.37	436.48
340	All Population	DALYs (Disability-Adjusted Life Years)	Singapore	Female	60+ years	Edentulism	Rate	2020	309.23	456.17	200.91
341	All Population	DALYs (Disability-Adjusted Life Years)	Malaysia	Female	60+ years	Edentulism	Rate	2020	823.22	1173.99	524.20
342	All Population	DALYs (Disability-Adjusted Life Years)	Cambodia	Female	60+ years	Edentulism	Rate	2021	647.07	935.13	411.51
343	All Population	DALYs (Disability-Adjusted Life Years)	Thailand	Female	60+ years	Edentulism	Rate	2012	615.99	893.59	393.58
344	All Population	DALYs (Disability-Adjusted Life Years)	Philippines	Female	60+ years	Edentulism	Rate	2012	982.28	1325.98	672.69

345	All Population	DALYs (Disability-Adjusted Life Years)	Lao People's Democratic Republic	Female	60+ years	Edentulism	Rate	2021	399.28	576.21	262.75
346	All Population	DALYs (Disability-Adjusted Life Years)	China	Female	60+ years	Edentulism	Rate	2018	573.91	808.38	387.59
347	All Population	DALYs (Disability-Adjusted Life Years)	Association of Southeast Asian Nations	Female	60+ years	Edentulism	Rate	2017	666.90	912.44	459.61
348	All Population	DALYs (Disability-Adjusted Life Years)	Myanmar	Female	60+ years	Edentulism	Rate	2018	400.18	580.58	256.70
349	All Population	DALYs (Disability-Adjusted Life Years)	Indonesia	Female	60+ years	Edentulism	Rate	2021	669.66	908.20	433.01
350	All Population	DALYs (Disability-Adjusted Life Years)	Viet Nam	Female	60+ years	Edentulism	Rate	2021	553.96	795.93	350.88
351	All Population	DALYs (Disability-Adjusted Life Years)	Singapore	Female	60+ years	Edentulism	Rate	2021	311.34	449.56	203.61
352	All Population	DALYs (Disability-Adjusted Life Years)	Malaysia	Female	60+ years	Edentulism	Rate	2021	819.29	1152.33	532.30
353	All Population	DALYs (Disability-Adjusted Life Years)	Brunei Darussalam	Female	60+ years	Edentulism	Rate	2018	256.51	373.82	167.26
354	All Population	DALYs (Disability-Adjusted Life Years)	Cambodia	Female	60+ years	Edentulism	Rate	2022	647.49	925.51	413.60
355	All Population	DALYs (Disability-Adjusted Life Years)	Lao People's Democratic Republic	Female	60+ years	Edentulism	Rate	2022	395.80	569.88	246.84
356	All Population	DALYs (Disability-Adjusted Life Years)	Indonesia	Female	60+ years	Edentulism	Rate	2022	667.49	909.15	432.12
357	All Population	DALYs (Disability-Adjusted Life Years)	Viet Nam	Female	60+ years	Edentulism	Rate	2022	551.70	780.36	355.56
358	All Population	DALYs (Disability-Adjusted Life Years)	Singapore	Female	60+ years	Edentulism	Rate	2022	313.01	450.78	202.70
359	All Population	DALYs (Disability-Adjusted Life Years)	Philippines	Female	60+ years	Edentulism	Rate	2013	984.83	1327.98	663.52
360	All Population	DALYs (Disability-Adjusted Life Years)	Malaysia	Female	60+ years	Edentulism	Rate	2022	815.68	1168.24	520.82
361	All Population	DALYs (Disability-Adjusted Life Years)	Thailand	Female	60+ years	Edentulism	Rate	2013	613.61	884.77	396.11
362	All Population	DALYs (Disability-Adjusted Life Years)	Indonesia	Female	60+ years	Edentulism	Rate	2023	660.54	895.01	426.56
363	All Population	DALYs (Disability-Adjusted Life Years)	Malaysia	Female	60+ years	Edentulism	Rate	2023	811.45	1166.59	518.46
364	All Population	DALYs (Disability-Adjusted Life Years)	Cambodia	Female	60+ years	Edentulism	Rate	2023	641.18	915.79	417.13
365	All Population	DALYs (Disability-Adjusted Life	Lao People's	Female	60+ years	Edentulism	Rate	2023	391.55	566.75	242.62

		Years)	Democratic Republic								
366	All Population	DALYs (Disability-Adjusted Life Years)	Viet Nam	Female	60+ years	Edentulism	Rate	2023	545.32	775.48	344.82
367	All Population	DALYs (Disability-Adjusted Life Years)	Singapore	Female	60+ years	Edentulism	Rate	2023	318.65	457.77	207.25
368	All Population	DALYs (Disability-Adjusted Life Years)	China	Female	60+ years	Edentulism	Rate	2019	611.90	857.30	412.43
369	All Population	DALYs (Disability-Adjusted Life Years)	Myanmar	Female	60+ years	Edentulism	Rate	2019	395.50	577.97	252.36
370	All Population	DALYs (Disability-Adjusted Life Years)	Association of Southeast Asian Nations	Female	60+ years	Edentulism	Rate	2018	661.57	900.88	455.09
371	All Population	DALYs (Disability-Adjusted Life Years)	Brunei Darussalam	Female	60+ years	Edentulism	Rate	2019	255.46	375.99	167.31
372	All Population	DALYs (Disability-Adjusted Life Years)	Philippines	Female	60+ years	Edentulism	Rate	2014	985.68	1338.44	653.41
373	All Population	DALYs (Disability-Adjusted Life Years)	Thailand	Female	60+ years	Edentulism	Rate	2014	611.38	868.71	396.27
374	All Population	DALYs (Disability-Adjusted Life Years)	Association of Southeast Asian Nations	Female	60+ years	Edentulism	Rate	2019	656.99	896.51	450.78
375	All Population	DALYs (Disability-Adjusted Life Years)	China	Female	60+ years	Edentulism	Rate	2020	623.41	869.78	420.30
376	All Population	DALYs (Disability-Adjusted Life Years)	Brunei Darussalam	Female	60+ years	Edentulism	Rate	2020	255.35	372.68	165.62
377	All Population	DALYs (Disability-Adjusted Life Years)	Myanmar	Female	60+ years	Edentulism	Rate	2020	389.52	555.04	245.53
378	All Population	DALYs (Disability-Adjusted Life Years)	Philippines	Female	60+ years	Edentulism	Rate	2015	982.66	1331.54	642.56
379	All Population	DALYs (Disability-Adjusted Life Years)	China	Female	60+ years	Edentulism	Rate	2021	625.96	869.39	422.01
380	All Population	DALYs (Disability-Adjusted Life Years)	Association of Southeast Asian Nations	Female	60+ years	Edentulism	Rate	2020	652.85	889.33	447.86
381	All Population	DALYs (Disability-Adjusted Life Years)	Thailand	Female	60+ years	Edentulism	Rate	2015	607.40	858.80	390.70
382	All Population	DALYs (Disability-Adjusted Life Years)	Myanmar	Female	60+ years	Edentulism	Rate	2021	387.80	557.68	247.49
383	All Population	DALYs (Disability-Adjusted Life Years)	Brunei Darussalam	Female	60+ years	Edentulism	Rate	2021	256.43	373.34	167.69
384	All Population	DALYs (Disability-Adjusted Life Years)	Association of Southeast Asian Nations	Female	60+ years	Edentulism	Rate	2021	649.80	888.49	443.26

385	All Population	DALYs (Disability-Adjusted Life Years)	China	Female	60+ years	Edentulism	Rate	2022	628.64	877.95	425.44
386	All Population	DALYs (Disability-Adjusted Life Years)	Myanmar	Female	60+ years	Edentulism	Rate	2022	384.53	556.83	250.64
387	All Population	DALYs (Disability-Adjusted Life Years)	Brunei Darussalam	Female	60+ years	Edentulism	Rate	2022	259.01	380.08	168.16
388	All Population	DALYs (Disability-Adjusted Life Years)	Association of Southeast Asian Nations	Female	60+ years	Edentulism	Rate	2022	647.56	883.67	442.22
389	All Population	DALYs (Disability-Adjusted Life Years)	China	Female	60+ years	Edentulism	Rate	2023	619.35	864.02	419.09
390	All Population	DALYs (Disability-Adjusted Life Years)	Myanmar	Female	60+ years	Edentulism	Rate	2023	382.28	557.36	249.04
391	All Population	DALYs (Disability-Adjusted Life Years)	Brunei Darussalam	Female	60+ years	Edentulism	Rate	2023	259.81	384.42	168.52
392	All Population	DALYs (Disability-Adjusted Life Years)	Philippines	Female	60+ years	Edentulism	Rate	2016	977.95	1327.32	637.37
393	All Population	DALYs (Disability-Adjusted Life Years)	Philippines	Female	60+ years	Edentulism	Rate	2017	973.38	1320.60	633.46
394	All Population	DALYs (Disability-Adjusted Life Years)	Thailand	Female	60+ years	Edentulism	Rate	2016	604.07	866.67	390.13
395	All Population	DALYs (Disability-Adjusted Life Years)	Thailand	Female	60+ years	Edentulism	Rate	2017	601.26	858.58	391.30
396	All Population	DALYs (Disability-Adjusted Life Years)	Association of Southeast Asian Nations	Female	60+ years	Edentulism	Rate	2023	641.96	877.90	439.35
397	All Population	DALYs (Disability-Adjusted Life Years)	Philippines	Female	60+ years	Edentulism	Rate	2018	969.22	1315.81	627.54
398	All Population	DALYs (Disability-Adjusted Life Years)	Thailand	Female	60+ years	Edentulism	Rate	2018	598.64	860.76	387.63
399	All Population	DALYs (Disability-Adjusted Life Years)	Philippines	Female	60+ years	Edentulism	Rate	2019	966.19	1315.17	625.12
400	All Population	DALYs (Disability-Adjusted Life Years)	Thailand	Female	60+ years	Edentulism	Rate	2019	595.34	857.37	384.18
401	All Population	DALYs (Disability-Adjusted Life Years)	Thailand	Female	60+ years	Edentulism	Rate	2020	592.08	851.08	376.17
402	All Population	DALYs (Disability-Adjusted Life Years)	Philippines	Female	60+ years	Edentulism	Rate	2020	961.11	1303.40	623.32
403	All Population	DALYs (Disability-Adjusted Life Years)	Philippines	Female	60+ years	Edentulism	Rate	2021	963.29	1307.06	621.65
404	All Population	DALYs (Disability-Adjusted Life Years)	Thailand	Female	60+ years	Edentulism	Rate	2021	592.77	860.60	383.25
405	All Population	DALYs (Disability-Adjusted Life Years)	Philippines	Female	60+ years	Edentulism	Rate	2022	957.04	1297.87	616.18

406	All Population	DALYs (Disability-Adjusted Life Years)	Thailand	Female	60+ years	Edentulism	Rate	2022	595.54	864.08	377.07
407	All Population	DALYs (Disability-Adjusted Life Years)	Philippines	Female	60+ years	Edentulism	Rate	2023	948.96	1289.16	612.03
408	All Population	DALYs (Disability-Adjusted Life Years)	Thailand	Female	60+ years	Edentulism	Rate	2023	592.45	857.74	374.64

Supplemental Table S6: Trend Projection of Periodontal Diseases from 2023 to 2033 - ASEAN

	Year	Value	Lower	Upper	Type
1	1990	147.65			Actual
2	1991	148.77			Actual
3	1992	149.59			Actual
4	1993	150.13			Actual
5	1994	150.39			Actual
6	1995	150.55			Actual
7	1996	149.70			Actual
8	1997	147.33			Actual
9	1998	144.38			Actual
10	1999	141.91			Actual
11	2000	140.79			Actual
12	2001	141.54			Actual
13	2002	143.64			Actual
14	2003	146.24			Actual
15	2004	148.45			Actual
16	2005	149.59			Actual
17	2006	150.00			Actual
18	2007	150.41			Actual
19	2008	150.75			Actual
20	2009	151.05			Actual
21	2010	151.37			Actual
22	2011	151.42			Actual
23	2012	151.31			Actual
24	2013	151.14			Actual
25	2014	151.13			Actual
26	2015	151.41			Actual
27	2016	152.13			Actual
28	2017	153.13			Actual
29	2018	154.12			Actual
30	2019	154.83			Actual
31	2020	155.19			Actual
32	2021	155.62			Actual
33	2022	156.22			Actual
34	2023	154.60			Actual
35	2024	150.48	131.73	153.10	Forecast
36	2025	146.10	131.52	156.28	Forecast
37	2026	143.02	131.94	159.07	Forecast
38	2027	141.88	132.26	161.05	Forecast
39	2028	142.42	132.06	162.14	Forecast
40	2029	143.90	131.32	162.53	Forecast
41	2030	145.51	149.53	151.43	Forecast
42	2031	146.66	143.04	149.15	Forecast

43	2032	147.10	137.28	148.77	Forecast
44	2033	146.93	133.46	150.30	Forecast

Supplemental Table S7: Trend Projection of Edentulism from 2023 to 2033 - ASEAN

	Year	Value	Lower	Upper	Type
1	1990	709.39			Actual
2	1991	706.30			Actual
3	1992	703.62			Actual
4	1993	700.91			Actual
5	1994	698.99			Actual
6	1995	697.62			Actual
7	1996	695.34			Actual
8	1997	693.39			Actual
9	1998	691.86			Actual
10	1999	691.68			Actual
11	2000	692.38			Actual
12	2001	693.11			Actual
13	2002	692.19			Actual
14	2003	690.83			Actual
15	2004	689.55			Actual
16	2005	689.74			Actual
17	2006	690.13			Actual
18	2007	689.70			Actual
19	2008	688.69			Actual
20	2009	686.46			Actual
21	2010	685.47			Actual
22	2011	683.27			Actual
23	2012	682.52			Actual
24	2013	682.45			Actual
25	2014	681.72			Actual
26	2015	677.78			Actual
27	2016	672.76			Actual
28	2017	666.90			Actual
29	2018	661.57			Actual
30	2019	656.99			Actual
31	2020	652.85			Actual
32	2021	649.80			Actual
33	2022	647.56			Actual
34	2023	641.96			Actual
35	2024	636.37	596.61	631.38	Forecast
36	2025	630.78	586.04	630.76	Forecast
37	2026	625.18	575.08	630.54	Forecast
38	2027	619.59	563.74	630.69	Forecast
39	2028	614.00	552.05	631.19	Forecast
40	2029	608.40	540.04	632.02	Forecast
41	2030	602.81	634.03	638.71	Forecast
42	2031	597.22	625.54	636.02	Forecast

43	2032	591.62	616.41	633.95	Forecast
44	2033	586.03	606.75	632.43	Forecast

Supplemental Table S8: Trend Projection of Periodontal Diseases from 2023 to 2033 - China

	Year	Value	Lower	Upper	Type
1	1990	170.99			Actual
2	1991	167.88			Actual
3	1992	165.54			Actual
4	1993	163.97			Actual
5	1994	163.15			Actual
6	1995	163.08			Actual
7	1996	164.22			Actual
8	1997	166.59			Actual
9	1998	169.36			Actual
10	1999	171.80			Actual
11	2000	173.21			Actual
12	2001	174.06			Actual
13	2002	175.01			Actual
14	2003	175.78			Actual
15	2004	176.01			Actual
16	2005	175.51			Actual
17	2006	172.05			Actual
18	2007	165.08			Actual
19	2008	157.01			Actual
20	2009	150.56			Actual
21	2010	147.76			Actual
22	2011	152.32			Actual
23	2012	163.23			Actual
24	2013	176.33			Actual
25	2014	187.28			Actual
26	2015	191.96			Actual
27	2016	188.94			Actual
28	2017	182.14			Actual
29	2018	174.88			Actual
30	2019	170.53			Actual
31	2020	168.86			Actual
32	2021	168.37			Actual
33	2022	167.34			Actual
34	2023	164.78			Actual
35	2024	161.44	157.31	181.38	Forecast
36	2025	159.13	163.27	187.59	Forecast
37	2026	159.59	167.04	191.87	Forecast
38	2027	163.35	166.63	193.90	Forecast
39	2028	169.35	162.90	193.31	Forecast
40	2029	175.43	158.04	190.53	Forecast
41	2030	179.45	159.85	163.03	Forecast
42	2031	180.27	154.66	163.59	Forecast

43	2032	178.10	151.65	167.52	Forecast
44	2033	174.28	152.63	174.08	Forecast

Supplemental Table S9: Trend Projection of Edentulism from 2023 to 2033 - China

	Year	Value	Lower	Upper	Type
1	1990	636.17			Actual
2	1991	629.68			Actual
3	1992	619.36			Actual
4	1993	605.19			Actual
5	1994	587.97			Actual
6	1995	568.69			Actual
7	1996	541.50			Actual
8	1997	506.62			Actual
9	1998	472.51			Actual
10	1999	447.24			Actual
11	2000	438.00			Actual
12	2001	448.23			Actual
13	2002	471.10			Actual
14	2003	499.63			Actual
15	2004	526.55			Actual
16	2005	544.61			Actual
17	2006	553.91			Actual
18	2007	561.18			Actual
19	2008	568.21			Actual
20	2009	568.16			Actual
21	2010	569.21			Actual
22	2011	553.37			Actual
23	2012	519.30			Actual
24	2013	478.38			Actual
25	2014	445.51			Actual
26	2015	430.79			Actual
27	2016	455.76			Actual
28	2017	512.77			Actual
29	2018	573.91			Actual
30	2019	611.90			Actual
31	2020	623.41			Actual
32	2021	625.96			Actual
33	2022	628.64			Actual
34	2023	619.35			Actual
35	2024	593.61	388.41	609.90	Forecast
36	2025	561.12	378.46	616.46	Forecast
37	2026	532.05	382.45	625.43	Forecast
38	2027	510.69	394.26	637.45	Forecast
39	2028	499.15	407.97	652.22	Forecast
40	2029	497.46	419.55	667.97	Forecast
41	2030	503.94	580.41	606.81	Forecast
42	2031	515.86	521.81	600.43	Forecast

43	2032	530.10	462.56	601.55	Forecast
44	2033	543.76	416.37	605.01	Forecast

Supplemental Table S10: Trend Projection of Periodontal Diseases from 2023 to 2033 - Indonesia

	Year	Value	Lower	Upper	Type
1	1990	176.03			Actual
2	1991	176.50			Actual
3	1992	176.90			Actual
4	1993	177.24			Actual
5	1994	177.41			Actual
6	1995	177.45			Actual
7	1996	177.64			Actual
8	1997	177.71			Actual
9	1998	177.61			Actual
10	1999	177.49			Actual
11	2000	177.34			Actual
12	2001	177.09			Actual
13	2002	176.84			Actual
14	2003	176.64			Actual
15	2004	176.48			Actual
16	2005	176.53			Actual
17	2006	176.76			Actual
18	2007	177.06			Actual
19	2008	177.45			Actual
20	2009	178.02			Actual
21	2010	178.68			Actual
22	2011	179.33			Actual
23	2012	180.26			Actual
24	2013	181.20			Actual
25	2014	182.20			Actual
26	2015	182.97			Actual
27	2016	183.55			Actual
28	2017	184.13			Actual
29	2018	184.55			Actual
30	2019	184.99			Actual
31	2020	185.36			Actual
32	2021	186.05			Actual
33	2022	185.75			Actual
34	2023	183.77			Actual
35	2024	184.00	182.55	187.33	Forecast
36	2025	184.24	182.55	187.79	Forecast
37	2026	184.47	182.58	188.24	Forecast
38	2027	184.71	182.62	188.67	Forecast
39	2028	184.94	182.67	189.09	Forecast
40	2029	185.17	182.73	189.49	Forecast
41	2030	185.41	182.93	185.07	Forecast
42	2031	185.64	182.72	185.75	Forecast

43	2032	185.88	182.62	186.32	Forecast
44	2033	186.11	182.57	186.84	Forecast

Supplemental Table S11: Trend Projection of Edentulism from 2023 to 2033 - Indonesia

	Year	Value	Lower	Upper	Type
1	1990	704.13			Actual
2	1991	700.44			Actual
3	1992	698.17			Actual
4	1993	696.42			Actual
5	1994	695.22			Actual
6	1995	695.45			Actual
7	1996	693.96			Actual
8	1997	693.40			Actual
9	1998	693.24			Actual
10	1999	694.27			Actual
11	2000	696.39			Actual
12	2001	701.97			Actual
13	2002	711.34			Actual
14	2003	721.64			Actual
15	2004	730.01			Actual
16	2005	733.70			Actual
17	2006	734.26			Actual
18	2007	734.35			Actual
19	2008	733.41			Actual
20	2009	731.19			Actual
21	2010	727.65			Actual
22	2011	723.37			Actual
23	2012	717.51			Actual
24	2013	710.80			Actual
25	2014	703.99			Actual
26	2015	697.52			Actual
27	2016	691.51			Actual
28	2017	685.58			Actual
29	2018	680.32			Actual
30	2019	676.82			Actual
31	2020	673.51			Actual
32	2021	669.66			Actual
33	2022	667.49			Actual
34	2023	660.54			Actual
35	2024	651.29	570.48	645.06	Forecast
36	2025	640.96	546.77	646.20	Forecast
37	2026	630.10	521.81	648.54	Forecast
38	2027	618.99	495.71	651.99	Forecast
39	2028	607.77	468.57	656.48	Forecast
40	2029	596.49	440.46	661.93	Forecast
41	2030	585.18	647.92	654.66	Forecast
42	2031	573.85	631.96	649.96	Forecast

43	2032	562.52	613.38	646.83	Forecast
44	2033	551.19	592.76	645.23	Forecast

Supplemental Table S12: Trend Projection of Periodontal Diseases from 2023 to 2033 - Malaysia

	Year	Value	Lower	Upper	Type
1	1990	704.13			Actual
2	1991	700.44			Actual
3	1992	698.17			Actual
4	1993	696.42			Actual
5	1994	695.22			Actual
6	1995	695.45			Actual
7	1996	693.96			Actual
8	1997	693.40			Actual
9	1998	693.24			Actual
10	1999	694.27			Actual
11	2000	696.39			Actual
12	2001	701.97			Actual
13	2002	711.34			Actual
14	2003	721.64			Actual
15	2004	730.01			Actual
16	2005	733.70			Actual
17	2006	734.26			Actual
18	2007	734.35			Actual
19	2008	733.41			Actual
20	2009	731.19			Actual
21	2010	727.65			Actual
22	2011	723.37			Actual
23	2012	717.51			Actual
24	2013	710.80			Actual
25	2014	703.99			Actual
26	2015	697.52			Actual
27	2016	691.51			Actual
28	2017	685.58			Actual
29	2018	680.32			Actual
30	2019	676.82			Actual
31	2020	673.51			Actual
32	2021	669.66			Actual
33	2022	667.49			Actual
34	2023	660.54			Actual
35	2024	651.29	570.48	645.06	Forecast
36	2025	640.96	546.77	646.20	Forecast
37	2026	630.10	521.81	648.54	Forecast
38	2027	618.99	495.71	651.99	Forecast
39	2028	607.77	468.57	656.48	Forecast
40	2029	596.49	440.46	661.93	Forecast
41	2030	585.18	647.92	654.66	Forecast
42	2031	573.85	631.96	649.96	Forecast

43	2032	562.52	613.38	646.83	Forecast
44	2033	551.19	592.76	645.23	Forecast

Supplemental Table S13: Trend Projection of Edentulism from 2023 to 2033 - Malaysia

	Year	Value	Lower	Upper	Type
1	1990	949.67			Actual
2	1991	939.92			Actual
3	1992	930.80			Actual
4	1993	925.29			Actual
5	1994	923.80			Actual
6	1995	923.17			Actual
7	1996	983.41			Actual
8	1997	1134.73			Actual
9	1998	1316.14			Actual
10	1999	1470.56			Actual
11	2000	1537.15			Actual
12	2001	1540.10			Actual
13	2002	1535.90			Actual
14	2003	1520.37			Actual
15	2004	1496.45			Actual
16	2005	1464.86			Actual
17	2006	1374.60			Actual
18	2007	1229.45			Actual
19	2008	1061.73			Actual
20	2009	919.33			Actual
21	2010	852.24			Actual
22	2011	846.81			Actual
23	2012	841.95			Actual
24	2013	838.63			Actual
25	2014	838.57			Actual
26	2015	838.05			Actual
27	2016	835.76			Actual
28	2017	828.92			Actual
29	2018	822.94			Actual
30	2019	820.33			Actual
31	2020	823.22			Actual
32	2021	819.29			Actual
33	2022	815.68			Actual
34	2023	811.45			Actual
35	2024	801.33	313.99	1238.24	Forecast
36	2025	790.81	220.51	1331.82	Forecast
37	2026	782.75	142.41	1411.96	Forecast
38	2027	778.00	76.97	1479.90	Forecast
39	2028	776.11	20.88	1538.10	Forecast
40	2029	776.17	-28.81	1589.15	Forecast
41	2030	777.19	760.35	842.31	Forecast
42	2031	778.44	662.37	919.25	Forecast

43	2032	779.49	543.29	1022.22	Forecast
44	2033	780.17	423.14	1132.85	Forecast

Supplemental Table S14: Trend Projection of Periodontal Diseases from 2023 to 2033 - Thailand

	Year	Value	Lower	Upper	Type
1	1990	156.89			Actual
2	1991	160.60			Actual
3	1992	163.23			Actual
4	1993	165.00			Actual
5	1994	166.01			Actual
6	1995	166.41			Actual
7	1996	164.97			Actual
8	1997	161.42			Actual
9	1998	157.16			Actual
10	1999	153.76			Actual
11	2000	152.37			Actual
12	2001	154.34			Actual
13	2002	159.55			Actual
14	2003	165.91			Actual
15	2004	171.57			Actual
16	2005	174.86			Actual
17	2006	176.35			Actual
18	2007	177.57			Actual
19	2008	178.63			Actual
20	2009	179.36			Actual
21	2010	180.02			Actual
22	2011	180.52			Actual
23	2012	180.95			Actual
24	2013	181.32			Actual
25	2014	181.79			Actual
26	2015	182.20			Actual
27	2016	182.97			Actual
28	2017	183.82			Actual
29	2018	184.69			Actual
30	2019	185.19			Actual
31	2020	185.39			Actual
32	2021	184.87			Actual
33	2022	185.80			Actual
34	2023	183.93			Actual
35	2024	180.50	154.24	188.62	Forecast
36	2025	176.78	151.59	192.36	Forecast
37	2026	173.75	150.29	196.00	Forecast
38	2027	171.94	149.77	199.17	Forecast
39	2028	171.43	149.53	201.66	Forecast
40	2029	171.98	149.17	203.44	Forecast
41	2030	173.14	178.56	182.43	Forecast
42	2031	174.47	171.63	181.93	Forecast

43	2032	175.59	164.57	182.92	Forecast
44	2033	176.31	158.58	185.30	Forecast

Supplemental Table S15: Trend Projection of Edentulism from 2023 to 2033 - Thailand

	Year	Value	Lower	Upper	Type
1	1990	667.09			Actual
2	1991	660.34			Actual
3	1992	653.99			Actual
4	1993	647.84			Actual
5	1994	643.95			Actual
6	1995	639.50			Actual
7	1996	636.30			Actual
8	1997	633.95			Actual
9	1998	632.32			Actual
10	1999	631.27			Actual
11	2000	631.15			Actual
12	2001	633.71			Actual
13	2002	634.91			Actual
14	2003	637.80			Actual
15	2004	640.31			Actual
16	2005	641.96			Actual
17	2006	639.71			Actual
18	2007	636.31			Actual
19	2008	631.47			Actual
20	2009	625.27			Actual
21	2010	623.00			Actual
22	2011	619.69			Actual
23	2012	615.99			Actual
24	2013	613.61			Actual
25	2014	611.38			Actual
26	2015	607.40			Actual
27	2016	604.07			Actual
28	2017	601.26			Actual
29	2018	598.64			Actual
30	2019	595.34			Actual
31	2020	592.08			Actual
32	2021	592.77			Actual
33	2022	595.54			Actual
34	2023	592.45			Actual
35	2024	589.46	559.54	596.79	Forecast
36	2025	586.56	553.32	597.54	Forecast
37	2026	583.72	547.29	598.13	Forecast
38	2027	580.93	541.46	598.56	Forecast
39	2028	578.17	535.79	598.84	Forecast
40	2029	575.43	530.30	598.97	Forecast
41	2030	572.71	585.80	593.13	Forecast
42	2031	570.01	579.19	593.93	Forecast

43	2032	567.32	572.52	594.92	Forecast
44	2033	564.63	565.95	595.90	Forecast
